# Lipid and Lipid Raft Alteration in Aging and Neurodegenerative Diseases: A Window for the Development of New Biomarkers

**DOI:** 10.3390/ijms20153810

**Published:** 2019-08-04

**Authors:** Fátima Mesa-Herrera, Lucas Taoro-González, Catalina Valdés-Baizabal, Mario Diaz, Raquel Marín

**Affiliations:** 1 Laboratory of Membrane Physiology and Biophysics, Department of Animal Biology, Edaphology and Geology; Faculty of Sciences, University of La Laguna, 38200 Sta. Cruz de Tenerife, Spain; 2 Laboratory of Cellular Neurobiology, Department of Basic Medical Sciences, Section of Medicine, Faculty of Health Sciences, University of La Laguna, 38200 Sta. Cruz de Tenerife, Spain; 3 Associate Research Unit ULL-CSIC “Membrane Physiology and Biophysics in Neurodegenerative and Cancer Diseases”, University of La Laguna, 38200 Sta. Cruz de Tenerife, Spain

**Keywords:** neurodegenerative diseases, lipids, lipid rafts, biomarkers, Alzheimer’s disease, Parkinson disease

## Abstract

Lipids in the brain are major components playing structural functions as well as physiological roles in nerve cells, such as neural communication, neurogenesis, synaptic transmission, signal transduction, membrane compartmentalization, and regulation of gene expression. Determination of brain lipid composition may provide not only essential information about normal brain functioning, but also about changes with aging and diseases. Indeed, deregulations of specific lipid classes and lipid homeostasis have been demonstrated in neurodegenerative disorders such as Alzheimer’s disease (AD) and Parkinson’s disease (PD). Furthermore, recent studies have shown that membrane microdomains, named lipid rafts, may change their composition in correlation with neuronal impairment. Lipid rafts are key factors for signaling processes for cellular responses. Lipid alteration in these signaling platforms may correlate with abnormal protein distribution and aggregation, toxic cell signaling, and other neuropathological events related with these diseases. This review highlights the manner lipid changes in lipid rafts may participate in the modulation of neuropathological events related to AD and PD. Understanding and characterizing these changes may contribute to the development of novel and specific diagnostic and prognostic biomarkers in routinely clinical practice.

## 1. Introduction

Lipids are biomolecules soluble in nonpolar organic solvents, usually insoluble in water, and are primarily known for their metabolic role in energy storage. Lipid species may differ depending on the configuration of their head group, the nature and number of carbon-carbon bonds, molecular weight, and whole structure [1,2]. In particular, the general lipid structure is determined by the comparative polarity of the head group with the tails, which are hydrophobic in nature. Biological lipids originate entirely or in part from two distinct types of biochemical subunits or “building-blocks”: ketoacyl and isoprene groups. Using biochemical approaches lipids can be sorted out in eight different classes, namely fatty acids, glycerolipids, glycerophospholipids (GPs), sphingolipids, sterols, prenols, saccharolipids, and polyketides [3]. 

Lipids are particularly abundant in the brain that is one of the fattiest organs in the body. Thus, in nerve cells, lipids represent 50–60% of cell membrane constituents. Among the most abundant lipid species identified in the central nervous system (CNS) are cholesterol, GPs, and sphingolipids [4]. Indeed, approximately 25% of the total content of cholesterol present in the human organism is localized in the brain. Noticeably, brain cholesterol comes almost entirely from the endogenous synthesis, being circulating cholesterol unable to cross the blood-brain barrier (BBB) [5]. In the adult brain, the major sterol is unesterified cholesterol but small amounts of demosterol and cholesteryl esters are also present. Furthermore, cholesterol is an essential structural component of cellular membranes and myelin. The majority of cholesterol (approximately 70–80%) is present in myelin sheaths formed by oligodendrocytes to insulate axons, the rest being part of neuronal and astrocyte membranes [6]. There is a highly efficient apo-E-dependent recycling of cholesterol in the brain including a potentially large turnover between neurons and glia cells [7]. Furthermore, brain cholesterol is a precursor of steroid hormones and neurosteroids [5]. Due to its hydrophobic nature, lipids not only are required as energy reservoirs, but they also play important structural and regulatory roles in specific and complex physiological functions. For instance, lipid molecules are related to cell transport, cell membrane formation, protein stabilization and modulation, cell signaling and transduction, neural communication, neurogenesis, synaptic transmission, signal transduction, membrane compartmentalization, and regulation of gene expression [8]. 

Fatty acids are also important structural components of neuronal membranes. These molecules are found as three main classes of esters: phospholipids, cholesterol esters and triglycerides. According to the aliphatic chain, fatty acids are classified in two types: saturated fatty acids that are fully composed by single bonds between neighbor carbons and unsaturated fatty acids with one or more carbon–carbon double-bonds. In addition, depending on the length of the carbon, fatty acids can be classified as short (<6 carbons), medium (6–12 carbons), long (14–22 carbons), and very long (>22 carbons) fatty acids. The main saturated fatty acids include palmitic acid (16:0), stearic acid (18:0) and the main monounsaturated is oleic acid (18:1) [9]. Fatty acids are synthetized in the liver and reach the brain from circulation after crossing the brain blood barrier (BBB) in a complex with albumin, lipoproteins and other carriers [10]. The mechanism of fatty acid import into the BBB is still uncertain. There are two prevailing hypotheses: (i) passive diffusion using a “flip-flop” mechanism independent of proteins [10,11,12] and (ii) protein-mediated transport mechanisms using specific fatty acid transport proteins, including caveolin-1 [13], intracellular fatty acid binding proteins 1–9 (FABP), and plasma membrane fatty acid binding protein (FABPpm) [14]. 

Approximately 50% of neuronal membrane is composed by polyunsaturated fatty acids (PUFAs), while in the myelin sheath these lipids constitute ~70% [15,16]. Two long and unsaturated fatty acids are considered to be essential and required in high amounts in nerve cells: arachidonic acid (AA) and docosahexaenoic acid (DHA) [5,8]. Indeed, DHA levels reach 15–50% of total fatty acids in neurons. Noticeably, the structural conformation of PUFAs provides particularly properties to the membrane milieu. Thus, the high number of double bonds in their molecules confer a dynamic microenvironment that guarantees membrane fluidity and plasticity [17]. Consequently, fatty acids perform various functions in the brain. These molecular moieties contribute to the structural integrity of cellular membranes, serving as signaling molecules, and, to a less extent, providing energy to cells. In particular, DHA and AA and are essential for brain accretion, synaptogenesis and neurogenesis [18]. 

Glycerophospholipids are the main phospholipids and constitute ~4.5–5% of whole brain wet weight and ~4.5% in gray matter [9]. They are classified as phosphatidylcholine (PC), phosphatidylethanolamine (PE), phosphatidylinositol (PI), phosphatidylserine (PS), and phosphatidylglycerol (PG) depending on the polar group. These phospholipids are esterified at positions *sn*-2 and *sn*-3 of the glycerol backbone with an unsaturated fatty acid (containing between one and six double bonds) and a saturated fatty acid, respectively. 

Another relevant lipid class in cell membranes is sphingolipids. These lipids bind to proteins forming lipid-protein complex that help to maintain a proper position and tight integration of proteins in lipid microenvironments. Also, sphingolipids are essential for the optimal function of ion channels and neuronal surface receptors, and their derived products are involved in the regulation of neuronal activity and gene expression [19]. Further, sphingolipids have a relevant role as precursor of lipid mediators and contribute to structural integrity of structural microdomains in neuronal membranes [20]. As structural components, they allow inter-leaflet association within the lipid bilayer due to their usual content of long-chain fatty acids, thereby leading to liquid-ordered domains. 

The combination of particular protein-bound lipid moieties embedded in the plasma membrane create highly organized multimolecular structures that lead to the organization of multiple and multidimensional levels of order [21]. In this sense, evidence accumulated over the last two decades indicate that cellular membranes exhibit structures called lipids rafts, liquid-ordered domains rich in sphingolipids, and cholesterol. Lipid rafts are also enriched in phosphatidylcholine, mainly with saturated acyl chains, although they also contain lower amounts of the other phospholipids, PE, PI, and PS. The predominant fatty acid is palmitate whereas unsaturated fatty acids are less represented in raft domains. The sphingolipids ganglioside 1 and 2 (GM1 and GM2, respectively) are highly abundant in these microstructures, and consequently are commonly used as typical lipid raft lipid markers. 

Lipid rafts function as multimolecular platforms where protein complexes interact to develop different physiological functions of which signaling regulation of signal transduction cascade are particularly relevant [22]. Raft resident proteins are often glycosylphosphatidylinositol (GPI) anchored proteins as well as scaffold proteins which include caveolins and flotillins [23]. The list of lipid raft-associated proteins is steadily increasing and belong to three categories: scaffolding proteins abundantly found in lipid rafts, proteins integrated into the liquid-disordered phase, and proteins shifting in and out these microdomains that represent an intermediate state [24,25]. Lipid raft-associated proteins include, among others, GPI-anchored receptors, receptor tyrosine kinases nonreceptor tyrosine kinases of the Src family, adapter and regulatory molecules of tyrosine kinase signaling cascade, heterotrimeric and small GTP-binding proteins, and cell adhesion molecules review in [14]. 

There is an increasing body of data indicating that lipid homeostasis in the nervous system is altered during physiological aging as well as in various neurodegenerative diseases such as Alzheimer’s disease (AD) and Parkinson’s disease (PD) [8]. Part of these aberrant changes has been related to lipid rafts observing alterations in their molecular lipid composition during aging and toxicity [26]. The modifications in the lipid raft organization may have important consequences in their physicochemical properties altering the local microenvironment. These alterations may contribute to rearrangements of raft integrated proteins [27] that may ultimately promote neurodegenerative processes in AD and PD [26]. 

In the following sections, we will discuss the data available on the importance of lipid homeostasis and lipid raft changes occurring with the progression of brain aging and age-related neuropathological events. 

## 2. Brain Lipids in Aging 

The physiological aging process includes changes at all levels of the biological organization that are counterbalanced by adaptive response mechanisms geared to preserving the composition and function within homeostatic thresholds. The adult human brain does not exhibit a uniform deterioration pattern of its structure and function as this organ appears to select vulnerable neuronal populations in parallel with injury [28]. The most functional decline associated with normal brain aging is caused by relative subtle changes such as the loss of dendrites, the reduction and morphological modifications of spines density and changes in the molecular profile of synapses [29]. All the described morphological anomalies are in correlation with the progressive and deleterious character of the aging process. 

Furthermore, the concentrations of most lipid species in the human brain have been shown to decrease after the age of 50 years old [9]. For instance, the percentage of dry matter in the human brain diminishes coupled to a decrease of nerve cell membrane lipids and a marked decline of total myelin content. Recent studies analyzing the lipid composition of different areas of human brain have confirmed the occurrence of age related lipid alterations. The first evidence correlating aging and affection of lipid profile of the human brain was reported by Burger & Seidel [30] and Rouser & Yamamoto [31]. Burger & Seidel [30] found that the brain lipid matrix suffers a significant throughout lifespan, observing an increase of total lipids during the first two decades of life, followed by a progressive decrease during adulthood and advanced aging. Rouser & Yamamoto [31] also observed a curvilinear regression of human brain lipid levels with age. However, in both studies, the lipid analysis was performed using whole brain tissue without any distinction between the gray and white matter. This fact may unmask the potential differences affecting distinct parts of the CNS. An additional parameter to take into consideration is the potential differences in lipid changes affecting distinct brain regions. Subsequent studies of different human brain areas confirmed the occurrence of age-related lipid changes. Thus, some evidence has reported the existence of region-dependent cholesterol changes, observing a reduction of cholesterol load in human frontal and temporal cortices as well as in the hippocampus, caudate nucleus, and cerebellum [32,33]. Moreover, cholesterol imbalances have been correlated with modifications in cholesterol synthesis and metabolism [34]. 

According to the literature, cholesterol is one of the most affected lipids in the brain during aging. It is widely accepted that cholesterol synthesis is very high in the developing brain, but this rate declines at low and constant levels during adulthood [35]. Most data reported point to a progressive reduction of cholesterol content in a wide variety of brain regions, including the human cortex, hippocampus and cerebellum, mouse synaptosomes, and cultured hippocampal [32,33,36]. In addition, cholesterol deficiency increases the vulnerability of hippocampal glia in primary culture to glutamate-induced excitotoxicity [37]. The detriment in cholesterol levels by aging also affects neurosteroids synthesis and amount. Neurosteroids, such as pregnenolone (PREG) and dehydroepiandrosterone (DHEA) are synthetized in the CNS and the peripheral nervous system, mainly, but not exclusively, in myelinating glial cells from cholesterol or steroid precursors imported from other parts of the body [38]. 

Several reports have demonstrated that besides cholesterol, other important lipids undergo a selective depletion with aging in both human brain and animal models. Indeed, brain aging also modifies phospholipid proportions. In particular, it have been observed a loss of approximately 10% in either PI, PE, or PC in the period between 40 and 100 years old [39]. This correlates with another study in which phospholipid concentrations were compared in different brain regions (gray matter, white matter, caudate nucleus, hippocampus, pons, cerebellum, and medulla oblongata). The results established a decrease of 10 to 20% of total phospholipid amount in 89–92-year-old individuals as compared to 33–36-year-old controls [32]. However, the phospholipid composition in other brain regions remained invariable. Furthermore, other data have reported that phospholipid reduction starts at the age of 20 and progressively becomes more pronounced by the age of 80, not observing significant differences between genders [33,40]. Overall, these data support the assumption that there is a progressive detriment in different lipid classes in brain lipid matrix as a result of aging, these variations becoming more significant after the age of 50 years old. 

Other lipid classes affected during aging are polyunsaturated fatty acids (PUFA). Some recent studies focused on the mitochondrial and microsomal lipidome have revealed a decrease of adrenic acid (22:4n-6) and AA (20:4n-6) (n-6 PUFAs) throughout adult life in the frontal and entorhinal cortices and hippocampus. These changes were mostly observed in the association of these n-6 PUFAs with PC, PE, and PS, whereas the amount of DHA (n-3 PUFA) remained invariable, or slightly decreased during the same period of time [41,42,43]. In line with these studies, another analysis of human brain fatty acid profile showed that there was a progressive decline in PUFA composition. This PUFA reduction was inversely correlated with stearoyl-CoA desaturase expression and transient compensatory elevations in elongase and desaturase gene expression [44]. Similar findings were found in rat brains, where aging caused variations in both sphingomyelin and the mono/polyunsaturated fatty acids ratio [45]. 

Regarding gangliosides, aging provokes a decline of neural glycolipids GD1a and GM1 levels and, to a lower extent, GT1b and GD1b in the human frontal cortex [46]. These observations correlated with the data obtained in rat cerebellar cortex levels both in vivo and in vitro [46]. However, only a moderate decrease of GD1a was shown in human hippocampus in correlation with aging, not detecting significant modifications in the level of other ganglioside classes [46]. Similar results were obtained in the visual cortex where there were virtually no changes in individual ganglioside species [46]. 

The reduction of cholesterol and other lipid classes in nerve cells may have important consequences from both the structural and functional points of view. Thus, in neuronal membranes it has been demonstrated that cholesterol changes reported with aging alter cell membrane fluidity, increasing their rigidity and physico-chemical properties [47]. Consequently, the cholesterol-related membrane structural impairments may affect the behavior of membrane-related proteins altering signaling transduction responses. This phenomenon may be particularly relevant in lipid rafts that are considered key points of signaling proteins clustered in signalosomes. In this order of ideas, we and others have reported anomalies in protein-protein interactions and protein multicomplex rearrangements due to changes of cholesterol and other lipid classes in lipid rafts, thereby modifying different transduction pathways that affect neuronal physiology [36,48,49,50,51]. In human studies, it has been reported that lipid composition in lipid rafts undergo significant alterations of specific lipid classes (cholesterol, sterol esters, PUFA, plasmalogens, sphingomyelin, sulfatides, and cerebrosides) and phospholipid-bound fatty acids (especially DHA and AA) in correlation with brain aging [52]. In agreement with this, some reports have found a reduction of n-3 and n-6 PUFA during physiological aging in different human brain areas (particularly in cortex and hippocampus) [44,53]. Interestingly, the impairment in the proportion of distinct lipid classes follows a gradual temporal context. For instance, saturated fatty acids appear to increase their levels in lipid rafts by the age of 70 whereas there is a decay in the main n-6 and n-3 PUFA (AA and DHA, respectively) by the age of 80 [54]. Nevertheless, the specific contribution of lipid patterns to the aging process remains to be fully characterized. Indeed, it has still not been clarified whether the changes in the confirmed lipids represent neutral changes with age, changes causing physiological aspects of aging, or rather are beneficial responses to damaging agents. It is interesting to note that even if modifications in the proportions of certain lipid classes have been reflected in the lipid raft milieu during neuropathology, these microdomains remain generally stable in healthy aged brains. For instance, a recent study carried out in our laboratory showed no significant changes in the mole percentage of total lipid classes and fatty acids from normal human frontal cortex throughout the lifespan (24–85 years) [36]. 

Overall, the data available indicate that both lipid homeostasis and metabolism are intimately associated with human brain aging. Taking into account that human brain performs a wide range of motor, sensory, regulatory, and cognitive functions which decline with advancing aging, it is plausible to hypothesize that regional alterations in human brain lipid matrix might be responsible for the pathophysiological processes involved in neurodegenerative diseases such as Alzheimer’s Disease (AD) and Parkinson’s Disease (PD). These issues are discussed in the next chapters.

### 2.1. Lipid and Lipid Raft Profile Alterations in Alzheimer’s Disease 

AD is a progressive neurodegenerative disease that represents approximately 70% of all cases of dementia diagnosed around the world. This pathology induces an irreversible neurological disorder that causes cognitive and behavioral impairment. AD symptomatology appears gradually and is characterized by a progressive detriment of cognitive abilities (such as language, memory, learning, and recognition abilities) as well as loss of motor skills. These features lead to changes in the behavior and personality, the inability to develop activities of daily living, and the loss of autonomy. 

Macroscopically, AD brains exhibit cortical atrophy (between 8 and 15% smaller than healthy brain) due to massive loss of neurons [55]. At the molecular level, β-amyloid peptide (Aβ), and tau protein have been identify as the main agents causing Alzheimer’s disease [56]. These two proteins have been studied intensively due to the formation of macroscopic aggregates, namely extracellular amyloid plaques and intracellular neurofibrillary tangles in AD brain tissue. In this sense, during the last decades, the amyloid cascade hypothesis [57] has been the most accepted scenario to explain the pathogenic processes of AD. According to the amyloid cascade hypothesis, Aβ oligomerization, and accumulation is a neurotoxic phenomenon considered a key factor of many pathological features of the disease [58,59,60]. Furthermore, senile plaques formation is accompanied by astrogliosis [61], neuroinflammation [62], and oxidative stress [63], being the neocortex and hippocampus the most affected brain areas [64]. In addition to macroscopic changes, AD brain also suffers biochemical changes including variations in calcium homeostasis and alterations in phospholipid and cholesterol metabolism that ultimately may modify mitochondrial functioning. It has been demonstrated that calcium dysregulation precedes and underlays other AD-related alterations such as oxidative stress, tau aberrant hyperphosphorylation and synaptic plasticity impairments [65]. Interestingly, these changes often appear early in the course of the disease, prior to plaque and tangle accumulation. The available data indicate that mitochondrial impairment associated with these molecular parameters may be related to alterations within a subdomain of the endoplasmic reticulum (ER) named mitochondria-associated ER membranes (MAM), which is an intracellular lipid raft-like domain (review in [66]). For instance, mitochondrial impairment produces a dysregulation of neuronal calcium homeostasis. In particular, Ca^2+^ dysregulation by the endoplasmic reticulum (ER) in AD mouse models results in augmented cytosolic Ca^2+^ levels, thereby triggering signaling cascades that are detrimental to neuronal function related with APP processing and accumulation of Aβ. [67]. Interestingly, experimental and computational studies have shown that Aβ oligomers increase permeability and open probability of calcium channels in the plasma membrane. These unspecific “pores” disrupt the homeostatic regulation of calcium balance increasing calcium influx into the cells that contributes to the calcium-induced alterations summarized above [68]. 

**Figure 1:**
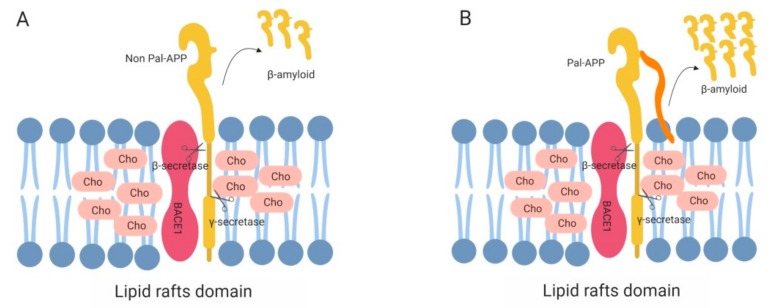
Schematic representation of amyloid precursor protein (APP) palmitoylation modulating both APP processing and Aβ generation in lipid rafts. (**A**). No APP palmitoylation. BACE1 (red) in lipid rafts cleaves APP at the β-cleavage site. Subsequent γ-secretase-mediated cleavage (γ) generates Aβ (orange). (**B**). APP palmitoylation. APP is palmitoylated, exhibiting a palmitic moiety (orange) attached to APP (pal-APP) that is recruited to lipid rafts. Our data suggest that palmitoylation may enhance BACE1-mediated cleavage of APP that may ultimately enhance generation of Aβ fragments and oligomerization. This and all the following figures were created with BioRender.com

The formation of Aα is triggered by the proteolytic processing of the amyloid precursor protein (APP), a transmembrane protein abundantly found in neurons. APP processing occurs in two distinct pathways: amyloidogenic and non-amyloidogenic processing. On the one hand, the non-amyloidogenic pathway involves α-secretase cleavage of APP. This cleavage occurs between Lys16 and Leu17, within the Aβ region [69] and precludes formation of Aβ. On the other hand, the amyloidogenic processing involves sequential cleavage of APP by β- and γ-secretases. This process ultimately releases Aβ peptide and a soluble ectodomain: sAPPβ [70]. Although APP itself is not a raft protein, a significant proportion of APP is localized in lipid rafts [71] following palmitoylation modification (Figure 1). Therefore, it is generally considered that APP cleavage is modulated within lipid raft microenvironments [2,72]. 

The particular protein/lipid composition is known to influence Aβ release [73] and aggregation [74]. Likewise, calcium homeostasis has also been related to the accumulation of α-amyloid oligomers as well as to the processing of APP (review in [75]). In this order of ideas, it has been suggested that cholesterol, a major component in lipid rafts, has different roles related to APP processing and Aβ production [76,77,78,79,80]. This lipid molecule plays an important role in the integration of APP within lipid rafts promoting its trafficking into these microdomains [81]. Wahrle et al. [79] observed that ϒ-secretase activity could be modulated following cholesterol fluctuations at the cell membrane. Thus, the activity of ϒ-secretase was abolished when the cholesterol levels decreased whereas this protein functionality was restored by replenishing the cholesterol reservoirs. The correlation between raft cholesterol content and ϒ-secretase levels also enhances the cleavage of APP depending on the availability of β-secretase (BACE1), thereby increasing Aβ production in lipid rafts [80]. BACE1 is located in rafts where it is stabilized by means of three palmitoylated residues in its structure [1,2]. Conversely, depletion of membrane cholesterol leads to an increase of α-secretase cleavage of APP [76,78]. Overall, these data reflect the importance of cholesterol homeostasis in the behavior of APP processing and Aα production, a fact that may have important consequences in AD. In support of this, some studies have reported alterations in cholesterol levels in specific brain areas in particular in regions with extensive Aβ deposits and neurofibrillary tangles (NFTs) related to AD. Cholesterol is synthesized in astrocytes and is transported from astrocytes to neurons bound to apoliprotein-E (ApoE). In close connection with AD, it has been solidly established the presence of ApoE4 allele as a primary genetic risk for sporadic AD. Indeed, the progression and development of the disease occurs in ~60% of cases of humans carrying this allele [82]. Amongst the brain regions more affected in ApoE4 carriers suffering AD are the middle frontal gyrus [83] and frontal cortex [84], whereas the cerebellum remained unaffected [83]. Furthermore, it has been demonstrated that membrane cholesterol deregulation allows the integration of Aβ peptide into nerve membranes [78]. Also, MAM serves as a regulatory hub for lipid regulation, in particular cholesterol and phospholipids [85]. Also, MAM has been observed as a regulatory core for lipid regulation of cholesterol trafficking. In this sense, Tambini and collaborates have shown that lipoproteins containing ApoE4 upregulated MAM function to a significantly greater degree than those containing ApoE3 [86]. 

Moreover, cholesterol derivatives may also take part of AD pathological scenario at the neuronal membrane. For instance, oxysterols are the oxidized derivate of cholesterol and are known to be involved in AD pathogenesis [87]. Two of these oxysterol molecules, 24-hydroxycholesterol (24-OH Chol) and 27- hydrocholesterol (27-OH Chol), have been found to increase BACE1 activity in human neuroblastoma SH-SY5Y cells. This phenomenon appears to be related to the ability of oxysterol to influence the cross-talk between two transcription factors, NF-kB and the growth arrest and DNA damage induced by gene 153 [88]. In addition, 24-hydroxycholesterol (24-OH Chol) and 27-hydrocholesterol have also been reported as modulators of Aβ aggregation and misfolding [89,90,91] leading to amyloid plaque deposition in AD. Within lipid rafts, fluctuations of cholesterol levels are considered a main impact factor of raft physico-chemical disturbances [92,93]. Conversely, Aβ oligomers can also perturb cholesterol levels in these microdomains, observing an inversely proportion of this lipid with Aβ presence [94]. 

Sphingomyelin is another major component of lipid rafts. In aged and AD brains, sphingomyelin metabolism experiments some changes leading to sphingomyelin decreased levels in parallel with an increase in the concentrations of ceramides which are sphingolipid-derived molecules [95]. Higher production of ceramide is due to an increase of sphingomyelinase (SMase) activity that hydrolyzes sphingomyelin. SMase activation generates sphingomyelin depletion, promoting an abnormal APP processing and cellular trafficking [96]. In addition, ceramide and ceramide 1-phosphate can act as lipid mediators that accumulate in AD brains. In brain cell models, it has been observed that ceramides accumulate in membranes by changing membrane viscosity and permeability [97,98] due to an activation of cytosolic phospholipase A2 (cPLA2) concomitantly with alterations in ion homeostasis and extrinsic apoptosis. In addition, the degradation products of cPLA2 metabolism are often proinflammatory [99]. Furthermore, Aβ accumulation, itself as a result of APP cleavage, activates SMase and mediates the cleavage of sphingomyelin thereby producing a toxic retroactive process. An important consequence of the unbalance production of this enzyme is the generation of a disproportion in protein–lipid ratio at the neuronal membrane that also affects signaling responses [100]. In agreement, some studies carried out with human blood samples have shown that alterations in the profile of sphingolipids also occur in patients with AD [95]. Indeed, the alterations in plasma sphingomyelin concentrations have been reported to correlate with cognitive decline occurring in AD patients [101]. Finally, other lipid modulators of SMase activity may also contribute to sphingolipid metabolism associated with AD-related molecular impairments. Thus, eicosanoids, docosanoids, and cannabinoids are key regulators of sphingolipid metabolism and signaling mediators of abnormal metabolism correlating with AD pathogenesis. 

Furthermore, lipid alterations observed in neuronal membranes during AD have also been related to a detriment of ganglioside metabolism. Thus, KO-ganglioside mice having a depletion of various types of gangliosides show a similar symptomatology to that developed in AD [102,103]. In this order of ideas, lipid profiles in AD patients reflect a detriment of ganglioside content in different brain areas associated with the pathogenesis of the disease [46]. Indeed, our own analyses using frontal cortices of AD patients have demonstrated significant alterations in gangliosides, sulfatides and cerebrosides in AD patients as compared with healthy subjects of similar age [36]. The disproportion of ganglioside levels in AD is also reflected in lipid rafts, where these lipid classes are particularly abundant. For instance, ganglioside GM-1 is a main component of these microdomains that has an important role in Aααaggregation [104,105,106]. In human neuroblastoma SH-SYS5 cells, GM-1 has been shown to interact with APP to enhance Aα processing through inhibition of α-cleavage [104]. Thus, this ganglioside acts as a linker and stabilizer of Aααaccumulation in lipid rafts through its hydrogen binding and electrostatic interaction with the peptide [73]. In support of this, it has been observed a ganglioside accumulation in senile plaques that enhances the conversion of Aβ in neurotoxic oligomers [107] and accelerates their formation [50,104]. Moreover, other ganglioside species may be involved in Aα formation. In this sense, it has been reported that activation of the expression of beta-1,4 N-acetylgalactosaminyltransferase 1 (B4GALNT1) (an enzyme that catalyzes the synthesis of GM2 and GD2 gangliosides) results in the increase of Aβ processing [108]. 

However, both sphingolipids and gangliosides integrated in lipid rafts may also act as neuroprotective agents reducing anti-inflammatory pathways and the impact of AD pathology [109]. The neuroprotective mechanisms of action of these lipid classes have not been fully characterized. The available data suggest that the protective roles of these lipids may take place through a variety of multiple and complementary strategies. For instance, in different models of excitotoxicity, it has been demonstrated that GM1 has the ability to regulate nuclear Ca^2+^ homeostasis [110]. In addition, GM1 may stimulate autophagy [111] thus participating in the clearance of potential toxic misfolded proteins [112]. 

**Figure 2:**
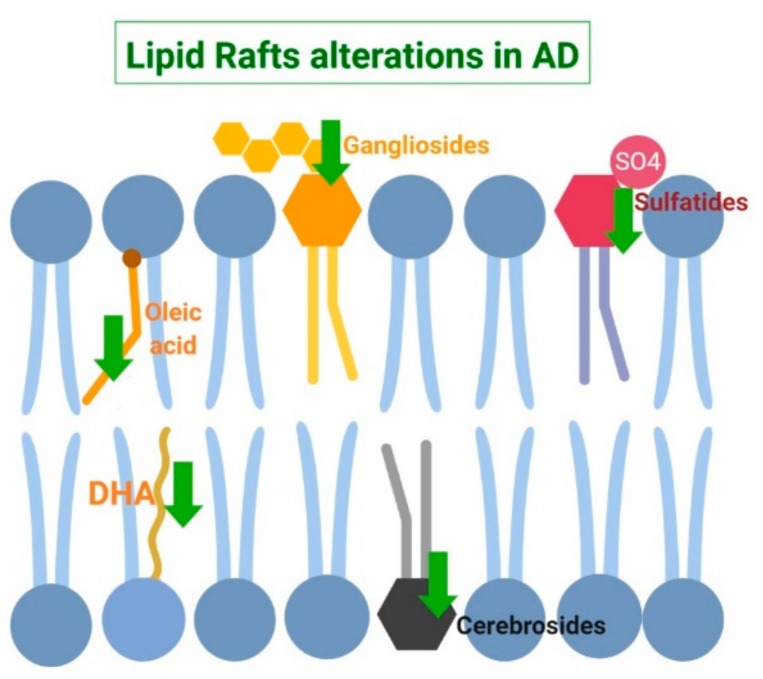
Schematic summary of lipid raft alterations in Alzheimer’s Disease (AD). In this pathologic state, both monounsaturated oleic acid (18:1) and polyunsaturated docosahexaenoic acid (DHA, (n-3 PUFA)) are depleted. Moreover, three member of the sphingolipids family are also reduced: gangliosides, cerebrosides, and sulfatides.

Other aberrant alterations in the lipid matrix of lipid rafts have been detected in different fatty acid species. Specifically, both monounsaturated fatty acids as well as n-3 long chain polyunsaturated fatty acids (n-3 PUFAS) appear to be reduced with respect to healthy controls [36]. Also, lipid rafts from AD brains display altered relationships between phospholipids and fatty acid [36] in AD brains. This disproportion may cause an increase in lipid rafts viscosity which may partially explain the aberrant signaling observed in AD [113]. Paralleling these changes, both higher molecular order and microviscosity in these domains have been demonstrated at early stages of AD [27,113]. The pathophysiological consequences of these events are the accumulation of β-secretase within lipid rafts thereby favoring the amyloidogenic processing of APP [27,114]. AD-induced modifications in fatty acid species as well as other lipid raft alterations have been summarized in Figure 2. 

Another interesting line of evidence suggests that changes in hormonal status may also affect brain lipid homeostasis during aging and AD. For instance, estrogen decline observed during menopause has been widely studied review in [107]. Indeed, postmenopausal reduction of estrogens not only affect lipid metabolism in liver and adipose tissue but also in the brain [52]. In this sense, using wild type and an AD model mice (APP/PS1), we have recently demonstrated that deprivation of estrogens affect lipid composition of hippocampal tissue as well as lipid related gene expression. The affected genes include those related to steryl/cholesteryl esters production (ACAT gene family) and those involved in de novo synthesis of cholesterol (HMGCoA gene), amongst others [115]. Furthermore, estrogen reduction causes the displacement of estrogen receptor (ER) from lipid rafts to non-raft fractions in brain samples from postmenopausal women. The alteration of this protein affects raft an ER-related multimolecular structure or signalosome with important consequences in estrogen signaling and neuroprotection [116]. 

### 2.2. Lipids and Lipid Raft Profile Alterations in Parkinson’s Disease

PD is the second most common neurodegenerative disease. PD is characterized by a progressive loss of dopaminergic neurons [117]. Age is the greatest risk factor for this disorder development. The prevalence and incidence increase nearly exponentially with age and rises a maximum after 80 years of age [118]. Gender is an established risk factor with an approximately 3:2 male:female ratio, respectively [119]. 

During the course of the disease, the patients suffer motor symptoms such as muscle rigidity, bradykinesia, rest tremor and postural and gait impairment [117]. Also, non-motor features are also frequently present in PD before the onset of the classical motor symptoms. These include impaired olfactory system, depression, rapid eye movement, visual hallucinations, behavior disorders, and mental decline [120]. Dementia is particularly prevalent in 83% of the patients affected by PD for more than 20 years [121]. The principal PD hallmark is the presence of intraneuronal inclusions called Lewy bodies formed by random and abnormal aggregation of α-synuclein protein. These aggregates are neurotoxic and cause loss of dopaminergic neurons in the substantia nigra pars compacta (SNpc) [122]. Current evidence indicates that Lewy bodies can be also formed extracellularly, increasing membrane permeability due to pore formation [123]. However, presently, it is still unclear the exact mechanisms by which α-synuclein aggregates and forms Lewy bodies in PD and other synucleinopathies. 

α-synuclein is a small, soluble protein expressed primarily in neurons, although it can also be detected in lower concentrations in other tissues. It is highly expressed throughout the mammalian brain and is enriched in presynaptic nerve terminals, where it associates with membranes and vesicular structures. Even though the exact physiological functions of α-synuclein are still unclear, but the presynaptic localization and its interaction with highly curved membranes and synaptic proteins strongly suggests a regulatory function associated in synapse processes. Thus, α-synuclein is involved in synaptic activity, synaptic plasticity, learning, neurotransmitter release, dopamine metabolism, synaptic vesicle pool maintenance, and/or vesicle trafficking review in [116].

Moreover, α-synuclein participates in lipid trafficking into the cell. For instance, α-synuclein has been reported to bind to fatty acids as a transport facilitator between the cytosol and membrane compartments [124]. In cultured cells, α-synuclein can regulate lipid metabolism by protecting lipid droplets from hydrolysis [125]. Also, a recent study has demonstrated that α-synuclein modulates the uptake of a fatty acids, in particular, palmitic acid (16:0) into neuronal membranes [126]. Furthermore, α-synuclein has been shown to induce membrane curvature and convert large vesicles into highly curved membrane tubules, cylindrical micelles and vesicles [127,128,129,130], driven by binding affinity, partition depth, and interleaflet order asymmetry [131]. In addition, α-synuclein has been reported to organize membrane components and modulate phospholipid composition [132]. This protein may also participate in lipid membrane homeostasis as a specific inhibitor of phospholipase D1 and D2 in vitro and in vivo [133,134]. However, the role of a-synuclein in lipid transport is still a subject of controversy that may be related to the particular local lipid structure in specific microenvironments. 

Furthermore, toxic nucleation and aggregation of α-synuclein at the plasma membrane may also depend on local lipid homeostasis [135]. In this order of ideas, α-synuclein accumulation is promoted by high levels of cholesterol and its oxidized products [136]. Previous studies have shown that specific oxysterols (e.g., 7β-OH-CHO and 24S-OH-CHO) at sub-lethal concentrations can regulate the transcription of α-synuclein and other genes involved in neuroinflammation and neurodegeneration [137,138,139,140,141]. Conversely, genetic deletion of α-synuclein in mice results in increased levels of cerebral cholesterol, cholesteryl esters and triacylglycerols [142]. 

Moreover, it has been demonstrated that α-synuclein can interact with n-3 PUFAs, such as α-linolenic, DHA, and eicosapentaenoic acid (EPA), forming a multimolecular complex configuration. In cultured cells, it has been observed an increase of α-synuclein insoluble aggregates in the presence of free PUFA [143,144]. In addition, a similar α-synuclein accumulation has been observed when the culture medium was supplemented with α-linolenic acid or EPA. Interestingly, this phenomenon appears to be specific of n-3 PUFA since α-synuclein aggregates were not detected upon culture treatment with either monounsaturated or saturated fatty acids [145]. In support of these observations, a study using PD transgenic mice has demonstrated that DHA-related oligomerization of α-synuclein takes place through the activation of proliferator-activated receptor (PPAR-γ) and retinoic X receptor (RXR) [146]. In a different order of ideas, lipid interaction with α-synuclein enhances the rate of oligomerization of the free protein and self-assembly in two human mutations linked to familial PD, A30P, and A53T mutations [142,143,144]. 

Other examples have demonstrated that anomalies in sphingolipid metabolism may also be involved in PD pathological progression. Thus, increased sphingomyelinase activity has been reported in PD brains, promoting an ceramide levels enhancement leading apoptosis [109]. Moreover, inhibition of sphingosine Kinase (Sphk1) enzyme-another enzyme in homeostasis regulation of sphingolipids-enhances α-synuclein secretion and propagation [147,148,149]. 

All these data suggest that the influence of lipid profile changes in nerve cell membranes involves multiple factors that may favor the optimal lipid microenvironment to enhance α-synuclein molecular rearrangements during aging and PD pathology. For instance, recent findings carried out in our research group in a mouse model of PD have demonstrated that membrane rearrangements of distinct lipid classes, such as gangliosides, PUFA, cholesterol, saturated fatty acids, and phospholipids have profound effects on α-syn distribution and oligomerization in abnormal structures in brain of PD mice model treated with 1-methyl-4-phenyl-1,2,3,6-tetrahydropyridine (MPTP) [150]. Specifically, it has been observed that GM1 ganglioside inhibits fibrillation of α-synuclein [151]. This observation is in line with previous studies claiming an implication of gangliosides in synucleinopathies [152]. In MPTP-mice, some anomalies were observed in the levels of other ganglioside species (GD1a, GD1b, and GT1b), particularly in aged mice [150]. Although the influence of GD1a, GD1b, and GT1b on α-synuclein membrane regulation has been rarely explored [153], these data suggest that distinct ganglioside classes may participate in the partitioning and conformational structure of α-synuclein. 

In human PD brains, lipid profile analyses have shown increased levels of AA and DHA in PD cortex compared to age-matched controls. Likewise, docosapentaenoic acid (22:5n-6) content and peroxidability index were augmented in PD cortex [154]. In parallel, PD gray matter exhibited lower levels of sphingolipids, cerebrosides, and sulfatides compared to age-matched control subjects, whereas levels of phospholipid classes and cholesterol were similar to those of control brains [154]. Another report comparing the frontal gray matter between control brains and PD has shown a reduction in sphingomyelin, sulfatides, and cerebrosides specifically in PD patients [154]. 

**Figure 3:**
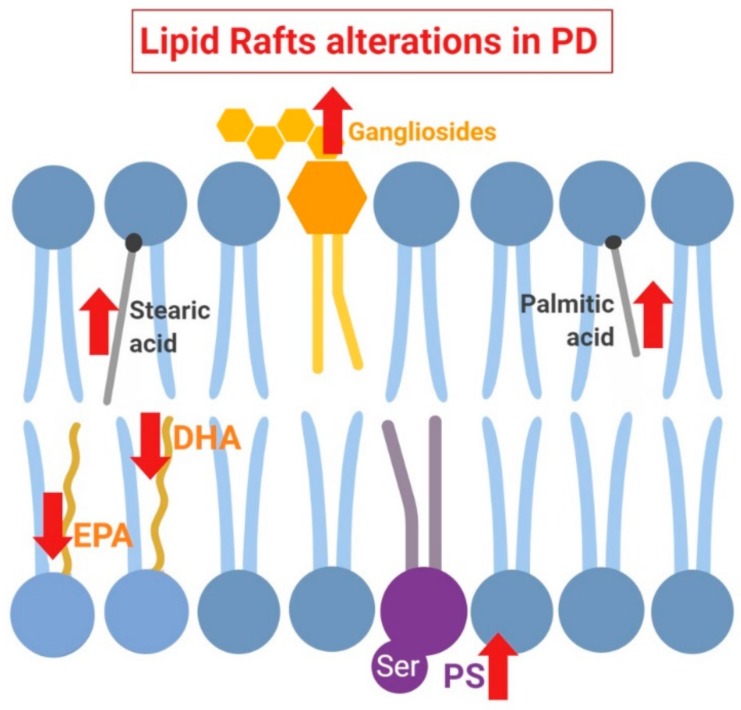
Schematic summary of lipid raft alterations in Parkinson’s Disease (PD). In this pathologic state, polyunsaturated docosahexaenoic acid (DHA) and eicosapentaenoic acid (EPA) are depleted, while levels of saturated fatty stearic and palmitic acids are increased. Furthermore, lipid raft content in gangliosides and phosphatidylserine (PS) is also pathologically augmented.

Taking into account the importance of local lipid microenvironment for α-synuclein configuration at the plasma membrane, it has been postulated that lipid rafts may play a crucial role in the dynamic and functionality of this protein at this level. For instance, α-synuclein appears to be integrated in this structures upon binding with phospholipids, preferentially phosphatidylserine [22,23]. This event also requires the presence of monounsaturated or polyunsaturated fatty acids, such as oleic acid or DHA, respectively, that are particularly represented in lipid rafts [155]. In support of α-synuclein specific binding to specific lipid species, some in vitro assays with artificial membrane models have demonstrated that α-synuclein binds preferentially to phosphatidylserine on the polyunsaturated acyl chain, indicating the coordinated recognition of head group in the context of specific side chain. Moreover, distinct n-3 and n-6 PUFA proportions appear to be crucial for α-synuclein conformation, a phenomenon that may stabilize the α-helix secondary structure of this protein [156]. For instance, depletion of DHA (n-3 PUFA) and AA (n-6 PUFA) in lipid rafts from human PD cortices [154] may have consequences in the dynamic of aggregation and fibrillation of α-synuclein facilitating the formation of neurotoxic aggregates [143]. Alterations in fatty acid species as well as other lipid raft alterations in PD have been summarized in Figure 3. 

Overall, these data indicate that PUFA proportion in lipid raft structure stability may play a significant role in α-synuclein configuration and homeostasis at the plasma membrane. Indeed, α-synuclein association with lipid raft moieties is a requirement for the synaptic localization, synaptic vesicular transporter and recycling of α-synuclein through PUFA associations [156]. In particular, α-synuclein/PUFA binding in lipid rafts seems to be a requirement contributing to synapse vesicle recycling following neuronal stimulation [157]. It has also been shown that α-synuclein interacts with lipid reservoir associated with scaffolding proteins such as caveolin-1 [158] and CD55 [159], suggesting a pleiotropic mechanism for this protein within membrane microdomains. 

On the other hand, our previous studies have demonstrated that instability of raft microdomains appears to be a crucial early event in the development of synucleinopathies such as PD [49,154]. In this sense, a plausible hypothesis is that the membrane lipid microenvironment may determine the modulation and trafficking of distinct α-synuclein molecular configurations. In MPTP mice as a model of PD neurotoxicity, we have previously demonstrated that rearrangement of membrane gangliosides, PUFA, cholesterol, saturated fatty acids, and phospholipids induced by brain aging and PD injury have profound effects on α-synuclein distribution and oligomerization in abnormal structures. Furthermore, these changes in protein trafficking may be extended to other biomarkers related to neurodegenerative diseases, such as the prion protein (PrPc), APP and glutamatergic receptors [150].

## 3. Lipids Alterations in Lipid Rafts as Potential Biomarkers for Neurodegenerative Diseases

The progressive increase of life expectancy in the population goes in parallel with a rise in the prevalence of the most common dementias such as AD and PD [160]. This phenomenon produces a socioeconomic hazard and an urgent public health challenge. Consequently, the establishment of accurate tools for early diagnosis and a follow-up in the progression of these diseases is crucial. Therefore, one of the main goals of the current clinical research is the identification of specific biomarkers for these neurodegenerative diseases in the aim of identifying the early signs of these diseases. 

Presently, the most frequently used clinical diagnostic items include neuropsychological, neuroimagery and biochemical tests. These approaches provide a good sensitivity and specificity to distinguish between patients suffering AD or PD from nondemented subjects although the available strategies are not sufficiently conclusive for the diagnosis at early asymptomatic stages [161]. In the case of AD, current diagnosis criteria include magnetic resonance imaging (MRI), metabolic changes detected by positron emission tomography (PET), and changes in the levels of Aβ and phosphorylated tau protein measured in the cerebrospinal fluid (CSF) [162]. 

In the case of PD, the diagnostic criteria include a comprehensive history and physical examination. Historically, pathological confirmation of the hallmark Lewy body on autopsy has been considered the criterion standard for diagnosis [163]. In clinical practice, diagnosis is typically based on the presence of a combination of cardinal motor features, associated and exclusionary symptoms and response to levodopa [164]. Although the diagnosis of PD is straightforward when patients have a classical presentation, differentiating PD from other forms of parkinsonism can be challenging early in the course of the disease, when signs and symptoms overlap with other syndromes [165]. From the biochemical point of view, there are not presently conclusive tests to confirm the diagnosis of PD. Consequently, a clinical diagnosis requires the clinician to review the patient’s history to assess symptoms and to discriminate from other alternative diagnoses, such as multiple-system atrophy, DLB disease, and essential tremor [166]. 

In the aim of developing new diagnosis and treatment strategies in the last decade, a promising field of study has focused on the search of novel biomarkers to detect molecular changes before the first neuropathological symptoms may appear. A biomarker is a molecular feature able to be quantified as an indicator of normal biological processes, pathogenic processes or pharmacological responses to therapeutic intervention. An ideal biomarker is characterized for its high specificity to predict the course of illness accurately and to reflect the degree of response to treatment. 

In parallel with genomic and proteomic approaches, the emerging field of lipidomics has allowed a new perspective into the disease paradigm. In this sense, due to lipid metabolism impairment occurring during neurodegeneration, it is plausible that fluctuations in targeted lipid species may be used as biomarkers for AD, PD and other synucleinopathies. Particularly relevant are the alterations of lipids and proteins interacting in lipid rafts that may be considered in the development of new diagnostic tools for neurodegenerative diseases. Interestingly, the available data indicate that lipid raft molecular changes and their derived metabolites can be monitored decades before the first neurodegenerative symptoms appear, and may be therefore used as predictors of ongoing pathologies. 

Novel molecular parameters reflecting raft lipid/protein fluctuations as well as other markers of inflammation, oxidative stress, and bioenergetic cell metabolism may be quantified through noninvasive methods such as CSF and peripheral blood in multiparametric tests. Presently, only a few studies have investigated lipid changes in human CSF. Han and collaborates [167] observed a 40% reduction in CSF levels of sulfatide in patients with a clinical dementia rating of 0.5 compared to controls. In this study, the authors suggested that the sulfatide/phosphatidylinositol ratio could be a useful marker in identifying patients with incident AD with a sensitivity of 90% and specificity of 100%. Other phospholipids are also modified in CSF. Thus, phosphocholine and sphingomyelin levels were shown to be increased at prodromal stages of AD as well as AD patients as compared to controls [39,168,169]. Sphingomyelin derivatives were also correlated with Aβ fragments and taun protein, whereas some sphingomyelin species also varied concomitantly with phospho-tau fluctuations [170]. Also, a recent cross-sectional study has reported that an 18-carbon acyl chain length ceramide was correlated with most of Aβ fragments and total tau, but not phospho-tau [39]. 

Other molecules related to lipid metabolism such as derivatives of oxysterol metabolism have also been studied as potential peripheral fluid markers for the pathogenesis of AD [171]. Thus, 24-OH cholesterol levels have been observed altered in AD patients, these variations being dependent on different stages of the disease [172]. Also, 27-OH cholesterol levels increase dramatically in the brain tissue of AD in different areas, in particular in frontal and occipital cortices [171]. Another potential detector of changes in lipid homeostasis may be related to quantification of lipid peroxidation products. Following this idea, different studies have evaluated in the levels of lipid peroxidation products as malondialdehyde (MDA), thiobarbituric acid-reactive substances (TBARS), isoprostane F2 (F_2_-IsoPs), and 4-hydroxinonenal in different human sample as blood, plasma, serum, urine and cerebrospinal fluid [173]. In particular, MDA has been evaluated in blood samples as a potential biomarker of AD. This molecule appears to have higher levels in the serum of AD patients as compared to healthy volunteers [174]. Interestingly, plasma concentrations of MDA were even more elevated in patients with mild cognitive impairment (MCI) than in AD subjects or healthy controls [175,176] as an indicative of the high lipid peroxidation scenario in early stages of this neuropathology. Similarly, 4-hydroxynonenal (4-HNE), an unsaturated moiety that is produced by lipid peroxidation in cells, appears to modify its production in AD patients according to the degree of cognitive impairment [176]. 

Another interesting candidate for AD detection may be the fatty acid-binding protein (FABP3). The levels of this protein appears significantly elevated in CSF from either early stages of AD or patients with clinical AD and concomitant cerebrovascular disease as compared with age-matched controls [177]. According to the available data, peripheral concentration of FABP3 discriminated between AD cases and healthy controls with a high sensitivity (76%) and significant specificity (84%) [178]. Moreover, FABP3 strongly correlated with both tau and phosphorylated tau related to AD [179]. Interestingly, the FABP3/Aβ42 ratio follows a similar trend to p-tau/Aβ42, suggesting that FABP3 may be an independent prognostic factor of the disease [180]. 

In the case of PD, the development of well-established biomarkers of the pathology remains challenging as the early molecular events have been poorly characterized. Nevertheless, significant progress has been performed in recent years to elucidate the initial causes of PD. From the pathological point of view, it seems that non-motor symptoms precede motor symptoms in contrast with the classical dogma [181]. Indeed, autopsy data indicate that pathological signs of PD are initiated in non-nigrostriatal neurons, suggesting the presence of early pathological events in non-motor brain areas prior to affections in the dopaminergic circuits [182]. 

A main hallmark under study is based upon the quantification of α-synuclein configuration in peripheral fluids [183,184]. However, the variations found in α-synuclein concentrations in the CSF do not provide unified criteria. Thus, several studies have found similar CSF total α-synuclein levels in PD patients and in controls [185,186,187,188,189], while other research has reported decreased CSF levels of α-synuclein in PD subjects [190,191,192,193,194,195]. Furthermore, even though the majority of recent studies have shown decreased α-synuclein levels in PD-CSF with respect to age-matched controls, similar protein downturn also appears in other synucleinopathies. For instance, Aerts y col. reported similar CSF α-synuclein levels in PD patients as compared with those suffering dementia with Lewy bodies, progressive supranuclear palsy and multiple system atrophy. These data led to the conclusion that α-synuclein variations may be a useful marker to distinguish synucleinopathic cases from control subjects but not to distinguish amongst distinct synucleinopathies [196]. 

Others promising candidates involved in lipid homeostasis and metabolism, oxidative stress influencing α-synuclein oligomerization are presently under study as potential PD diagnostic tools detectable in CSF [197]. As an example, endocannabinoids are a group of lipids derivate of monoacylglicerol (MAG) linked with PD disease [198]. Two studies have reported high levels of anandamide (AEA), a type of endocannabinoid, in the CSF of untreated PD patients that were restored upon dopamine treatment [199,200]. This finding suggests that AEA may act as a monitoring biomarker of PD progress. Moreover, some lipid carriers may be potential candidates to be monitored during PD development. In this sense, Apo1A is an apolipoprotein that participates in lipid transport in brain in cooperation with ApoE [201,202]. Detriment of Apo1A serum levels correlates with less efficient transport of high density lipoproteins, ultimately reducing brain cholesterol homeostasis and function. The fact that Apo1A concentrations show lower levels in CSF from PD patients indicates a deficit in cholesterol balance, a parameter that may be detected in peripheral CSF as a biomarker of PD progression [203,204,205]. 

Oxidative stress also occurs during the course of PD enhancing biological damage. For instance, oxidative stress is at the basis of decreased levels of both PI and PC phospholipids [204]. Similar to AD, some lipid peroxidative metabolites such as MDA were shown to be increased in PD brains, in particular in the substantia nigra pars compacta (SNpc) as compared with other Parkinsonian brain regions [205]. The levels of HNE are also increased in dopaminergic cells of SNpc and in peripheral fluids from PD patients [206,207,208]. Also, reactive oxygen species (ROS), such as oxide 8-hydroxyguanine (8-OHG) and its derivative 8-hydroxy-2’-deoxyguanosine (8-OHdG), increase their levels in the serum of PD patients compared with healthy individuals [209,210,211], thereby representing another group of potential biomarkers for PD. Overall, these findings suggest that a multiparametric analysis of distinct molecular species associated with lipid peroxidation and oxidative stress may be good candidates as CSF biomarkers in PD diagnosis. Indeed, the in-depth study of these alterations as a strategy to develop possible new diagnostic tools is paramount to improve clinical diagnosis of synucleinopathies. Table 1 summarizes the distinct lipid species changes observed in both human brain and peripheral tissues during AD and PD development. 

## 4. Conclusions

The brain lipidome is vast and complex. The variety of lipid species in this organ plays key functions ranging from activation of neurogenesis and synaptogenesis to modulation of synaptic transmission. Since lipids are responsible of a huge number of physiological functions in the brain, their altered metabolism is consequently a potential reflect of brain dysfunctioning. Therefore, new advances to better characterize the brain lipidome as well as the exploration of the specific role of targeted lipid species are a matter of intense research. In this sense, membrane lipid microstructures such as lipid rafts may be particularly relevant to explore the early events of age-dependent neurodegenerative diseases. In particular in neurons, early lipid impairment of these microdomains are key factors affecting the conformation and intracellular signaling of raft integrated proteins involved in pathological events. Therefore, the characterization of lipid raft patterns in different brain areas may be a key point to identify early neurodegeneration events, even in asymptomatic stages. These analyses may provide new hints for the development of measurable multiparametric tools of early diagnosis of neurodegeneration. Furthermore, determination of brain lipid alterations and their effects on lipid/protein and protein/protein associations may provide a hint in the research of potential pharmacological targets for clinical early intervention of these devastating disorders. 

## Figures and Tables

**Table 1:** Changes of lipid species in human bran and peripheral fluids under Alzheimer’s disease or Parkinson’s disease.

Distinct Changes of Lipid Species in Human Brain and Peripheral Fluids			
Alzheimer’s Disease			
Lipid	Variation	Sample	Reference
Ceramide	Increase	Brain tissue	[209]
Sphingomyelin	Decrease	Brain tissue	[209]
Phosphatidylcholine	Decrease	Serum	[210]
Phosphatidylinositol	Decrease	Serum	[210]
Phosphatidylethanolamine	Decrease	Serum	[210]
DHA	Decrease	Lipid rafts	[36]
Linolenic acid	Decrease	Plasma	[211]
ARA	Increase	Brain tissue	[212]
Palmitic acid	Increase	Brain tissue	[213]
Stearic acid	Increase	Brain tissue	[213]
Oleic acid	Decrease	Lipid rafts	[36]
Gangliosides	Decrease	Brain tissue/Lipid rafts	[36,46]
Cerebrosides	Decrease	Lipid rafts	[36]
Sulfatides	Decrease	Lipid rafts	[36]
**Lipid Metabolites**			
24-OH	Increase	Brain tissue	[172]
27-OH	Increase	Brain tissue	[171]
**Oxidative Stress Markers**			
MDA	Increase	Serum/Plasma	[174,175,214]
4-HNE	Increase	Brain tissue	[176]
**Proteins Related with Lipid Metabolism**			
HDL	Decrease	Serum	[77]
LDL	Increase	Serum	[77]
FABP3	Increase	CSF	[177]
**Parkinson’s Disease**			
**Lipids**	**Variation**	**Sample**	**Reference**
Ceramide	Increase	Brain tissue	[109]
Sphingomyelin	Decrease	Brain tissue	[215]
Phosphatidylcholine	Decrease	Brain tissue	[216]
Phosphatidylserine	Increase	Lipid rafts	[154]
Phosphatidylinositol	Decrease	Brain tissue	[204]
Phosphatidylethanolamine	Decreased	Brain tissue	[215]
EPA	Decrease	Lipid rafts	[49,154]
DHA	Decrease	Lipid rafts	[49,154]
Palmitic acid	Increase	Lipid rafts	[154]
Stearic acid	Increase	Lipid rafts	[154]
Oleic acid	Decrease	CSF	[217]
Palmitoleic acid	Decrease	CSF	[217]
Linoleic acid	Decrease	CSF	[217]
Gangliosides	Increase	Lipid rafts	[150]
**Lipid Metabolites**			
Endocannabinoids	Increase	CSF	[199,200]
Apo1	Decrease	CSF	[204,205]
**Oxidative Stress Markers**			
MDA	Increase	Brain tissue	[205]
4-HNE	Increase	SNPc/CSF	[206]
8-OHdG	Increase	Serum/CSF	[209,210,211]

## Abbreviations

24-OH Cho	24-hydroxycholesterol
27-OH Cho	27- hydroxycholesterol
8-OHdG	8-hydroxyguanosine oxized
4-HNE	4-hydroxynonenal
8-OHG	8-hydroxyguanine
AA	Arachidonic acid
AD	Alzheimer’s Disease
ALA	Linolenic acid
Aβ	β-amyloid peptide
B4GALNT1	beta-1,4 N-acetylgalactosaminyltransferase 1
CNS	Central nervous system
cPLA2	Cytosolic phospholipase A2
CSF	Cerebrospinal fluid
DHA	Docosahexaenoic acid
DHEA	Dehydroepiandrosterone
DLBD	Dementia with Lewy bodies disease
EPA	Eicosapentaenoic acid
FABP	Fatty acid-binding protein
GM1	Ganglioside 1
GM2	Ganglioside 2
GPI	Glycosylphosphatidylinositol
LA	Linoleic acid
LB	Lewy Body
MDA	Malondialdehyde
MCI	Mild cognitive impairment
NFTs	Neurofibrillary tangles
PC	phosphatidylcholine
PD	Parkinson’s Disease
PE	Phosphatidylethanolamine
PG	Phosphatidylglycerol
PI	Phosphatidylinositol
PREG	Pregnenolone
PS	Phosphatidylserine
PUFA	Polyunsaturated fatty acid
ROS	Reactive oxygen species
TBARS	Thiobarbituric acid-reactive substances
sAPPβ	Soluble amyloid precursor protein
SMase	Sphingomyelinase
SNpc	substantia nigra pars compacta
*Sphk1*	Sphingosine Kinase
PSP	Progressive supranuclear palsy
MSA	Multiple system atrophy
HDL	High density lipoprotein

## References

[1] Kalvodova L., Kahya N., Schwille P., Ehehalt R., Verkade P., Drechsel D., Simons K. (2005). Lipids as Modulators of Proteolytic Activity of BACE: Involvement of Cholesterol, Glycosphingolipids, and Anionic Phospholipids in Vitro. J. Biol. Chem..

[2] Vetrivel K.S., Thinakaran G. (2010). Membrane Rafts in Alzheimer’s Disease Beta-Amyloid Production. Biochim. Biophys. Acta—Mol. Cell Biol. Lipids.

[3] Spener F., Dennis E.A., Raetz C.R.H., Nishijima M., Subramaniam S., van Meer G., Shimizu T., Wakelam M.J.O., Murphy R.C., Fahy E. (2008). Update of the LIPID MAPS Comprehensive Classification System for Lipids. J. Lipid Res..

[4] Cermenati G., Mitro N., Audano M., Melcangi R.C., Crestani M., De Fabiani E., Caruso D. (2015). Lipids in the Nervous System: From Biochemistry and Molecular Biology to Patho-Physiology. Biochim. Biophys. Acta—Mol. Cell Biol. Lipids.

[5] Saeed A.A., Genové G., Li T., Lütjohann D., Olin M., Mast N., Pikuleva I.A., Crick P., Wang Y., Griffiths W. (2014). Effects of a Disrupted Blood-Brain Barrier on Cholesterol Homeostasis in the Brain. J. Biol. Chem..

[6] Di Scala C., Troadec J.D., Lelièvre C., Garmy N., Fantini J., Chahinian H. (2014). Mechanism of Cholesterol-Assisted Oligomeric Channel Formation by a Short Alzheimer β-Amyloid Peptide. J. Neurochem..

[7] Varilly P., Chandler D. (2012). APOE and Cholesterol in Aging and Disease in the Brain. J. Stat. Phys..

[8] Wong M.W., Braidy N., Poljak A., Pickford R., Thambisetty M., Sachdev P.S. (2017). Dysregulation of Lipids in Alzheimer’s Disease and Their Role as Potential Biomarkers. Alzheimer’s Dement..

[9] Naudí A., Cabré R., Jové M., Ayala V., Gonzalo H., Portero-Otín M., Ferrer I., Pamplona R. (2015). Lipidomics of Human Brain Aging and Alzheimer’s Disease Pathology. Int. Rev. Neurobiol..

[10] Hamilton J.A., Brunaldi K. (2007). A Model for Fatty Acid Transport into the Brain. J. Mol. Neurosci..

[11] Hamilton J.A., Guo W., Kamp F. (2002). Mechanism of Cellular Uptake of Long-Chain Fatty Acids: Do We Need Cellular Proteins?. Mol. Cell. Biochem..

[12] Mitchell R.W., Hatch G.M. (2011). Fatty Acid Transport into the Brain: Of Fatty Acid Fables and Lipid Tails. Prostaglandins Leukot. Essent. Fat. Acids.

[13] Trigatti B.L., Anderson R.G.W., Gerber G.E. (1999). Identification of Caveolin-1 as a Fatty Acid Binding Protein. Biochem. Biophys. Res. Commun..

[14] Mitchell R.W., On N.H., Del Bigio M.R., Miller D.W., Hatch G.M. (2011). Fatty Acid Transport Protein Expression in Human Brain and Potential Role in Fatty Acid Transport across Human Brain Microvessel Endothelial Cells. J. Neurochem..

[15] Hooijmans C.R., Kiliaan A.J. (2008). Fatty Acids, Lipid Metabolism and Alzheimer Pathology. Eur. J. Pharmacol..

[16] Fester L., Zhou L., Bütow A., Huber C., Von Lossow R., Prange-Kiel J., Jarry H., Rune G.M. (2009). Cholesterol-Promoted Synaptogenesis Requires the Conversion of Cholesterol to Estradiol in the Hippocampus. Hippocampus.

[17] Hadders-Algra M. (2008). Prenatal Long-Chain Polyunsaturated Fatty Acid Status: The Importance of a Balanced Intake of Docosahexaenoic Acid and Arachidonic Acid. J. Perinat. Med..

[18] Ruipérez V., Darios F., Davletov B. (2010). Alpha-Synuclein, Lipids and Parkinson’s Disease. Prog. Lipid Res..

[19] Tillman T.S., Cascio M. (2003). Effects of Membrane Lipids on Ion Channel Structure and Function. Cell Biochem. Biophys..

[20] Berkecz R., Kovács Z., Penke B., VÍgh L., Crul T., Paragi G., Gera J. (2018). The Role of Lipids and Membranes in the Pathogenesis of Alzheimer’s Disease: A Comprehensive View. Curr. Alzheimer Res..

[21] Sonnino S., Prinetti A. (2012). Membrane Domains and the “Lipid Raft” Concept. Curr. Med. Chem..

[22] Dart C. (2010). Lipid Microdomains and the Regulation of Ion Channel Function. J. Physiol..

[23] Michel V., Bakovic M. (2007). Lipid Rafts in Health and Disease. Biol. Cell.

[24] Levental I., Grzybek M., Simons K. (2010). Greasing Their Way: Lipid Modifications Determine Protein Association with Membrane Rafts. Biochemistry.

[25] Pike L.J. (2008). The Challenge of Lipid Rafts. J. Lipid Res..

[26] Marin R., Fabelo N., Fernández-Echevarría C., Canerina-Amaro A., Rodríguez-Barreto D., Quinto-Alemany D., Mesa-Herrera F., Díaz M. (2016). Lipid Raft Alterations in Aged-Associated Neuropathologies. Curr. Alzheimer Res..

[27] Díaz M., Fabelo N., Martín V., Ferrer I., Gómez T., Marín R. (2014). Biophysical Alterations in Lipid Rafts from Human Cerebral Cortex Associate with Increased BACE1/AβPP Interaction in Early Stages of Alzheimer’s Disease. J. Alzheimer’s Dis..

[28] Wang X., Michaelis M.L., Michaelis E.K. (2010). Functional genomics of brain aging and Alzheimer’s disease: focus on selective neuronal vulnerability. Curr. Genom..

[29] Mattson M.P., Magnus T. (2006). Ageing and Neuronal Vulnerability. Nat. Rev. Neurosci..

[30] Burger M., Seidel K. (1958). Chemical Biomorphosis of the Human Brain and Sciatic Nerve; a Survey. Z. Alternsforsch..

[31] Rouser G., Yamamoto A. (1968). Curvilinear Regression Course of Human Brain Lipid Composition Changes with Age. Lipids.

[32] Söderberg M., Edlund C., Kristensson K., Dallner G. (1990). Lipid Compositions of Different Regions of the Human Brain During Aging. J. Neurochem..

[33] Svennerholm L., Boström K., Helander C.G., Jungbjer B. (1991). Membrane Lipids in the Aging Human Brain. J. Neurochem..

[34] Thelen K.M., Falkai P., Bayer T.A., Lütjohann D. (2006). Cholesterol Synthesis Rate in Human Hippocampus Declines with Aging. Neurosci. Lett..

[35] Dietschy J.M., Turley S.D. (2004). Thematic Review Series: Brain Lipids. Cholesterol Metabolism in the Central Nervous System during Early Development and in the Mature Animal. J. Lipid Res..

[36] Martín V., Fabelo N., Santpere G., Puig B., Marín R., Ferrer I., Díaz M. (2010). Lipid Alterations in Lipid Rafts from Alzheimer’s Disease Human Brain Cortex. J. Alzheimer’s Dis..

[37] Chou Y., Lin S., Hsin L., Tsai H., Mei C. (2003). Cholesterol Deficiency Increases the Vulnerability of Hippocampal Glia in Primary Culture to Glutamate-Induced Excitotoxicity. Neurochem. Int..

[38] Brinton R.D. (2013). Neurosteroids as Regenerative Agents in the Brain: Therapeutic Implications. Nat. Publ. Gr..

[39] Farooqui A.A., Liss L., Horrocks L.A. (1988). Neurochemical Aspects of Alzheimer’s Disease: Involvement of Membrane Phospholipids. Metab. Brain Dis..

[40] Svennerholm L., Boström K., Jungbjer B., Olsson L. (2010). Membrane Lipids of Adult Human Brain: Lipid Composition of Frontal and Temporal Lobe in Subjects of Age 20 to 100 Years. J. Neurochem..

[41] Norris S.E., Friedrich M.G., Mitchell T.W., Truscott R.J.W., Else P.L. (2015). Human Prefrontal Cortex Phospholipids Containing Docosahexaenoic Acid Increase during Normal Adult Aging, Whereas Those Containing Arachidonic Acid Decrease. Neurobiol. Aging.

[42] Hancock S.E., Friedrich M.G., Mitchell T.W., Truscott R.J.W., Else P.L. (2015). Decreases in Phospholipids Containing Adrenic and Arachidonic Acids Occur in the Human Hippocampus over the Adult Lifespan. Lipids.

[43] Else P.L., Hancock S.E., Friedrich M.G., Mitchell T.W., Truscott R.J.W. (2017). The Phospholipid Composition of the Human Entorhinal Cortex Remains Relatively Stable over 80 Years of Adult Aging. GeroScience.

[44] Rider T., Tso P., Liu Y., Jandacek R., McNamara R.K. (2008). The Aging Human Orbitofrontal Cortex: Decreasing Polyunsaturated Fatty Acid Composition and Associated Increases in Lipogenic Gene Expression and Stearoyl-CoA Desaturase Activity. Prostaglandins Leukot. Essent. Fat. Acids.

[45] Venable M.E., Webb-Froehlich L.M., Sloan E.F., Thomley J.E. (2006). Shift in Sphingolipid Metabolism Leads to an Accumulation of Ceramide in Senescence. Mech. Ageing Dev..

[46] Kracun I., Rosner H., Drnovsek V., Vukelic Z., Cosovic C., Trbojevic-Cepe M., Kubat M. (1992). Gangliosides in the Human Brain Development and Aging. Neurochem. Int..

[47] Vanmierlo T., Lütjohann D., Mulder M. (2011). Brain Cholesterol in Normal and Pathological Aging. OCL—Ol. Corps Gras Lipides.

[48] Colin J., Gregory-Pauron L., Lanhers M.C., Claudepierre T., Corbier C., Yen F.T., Malaplate-Armand C., Oster T. (2016). Membrane Raft Domains and Remodeling in Aging Brain. Biochimie.

[49] Marin R., Fabelo N., Martín V., Garcia-Esparcia P., Ferrer I., Quinto-Alemany D., Díaz M. (2017). Anomalies Occurring in Lipid Profiles and Protein Distribution in Frontal Cortex Lipid Rafts in Dementia with Lewy Bodies Disclose Neurochemical Traits Partially Shared by Alzheimer’s and Parkinson’s Diseases. Neurobiol. Aging.

[50] Yamamoto N., Igbabvoa U., Shimada Y., Ohno-iwashita Y., Kobayashi M., Wood W.G., Fujita S.C., Yanagisawa K. (2004). Accelerated Aβ Aggregation in the Presence of GM1-Ganglioside-Accumulated Synaptosomes of Aged ApoE4-Knock-in Mouse Brain. FEBS Lett..

[51] Echeverría F., Valenzuela R., Catalina Hernandez-Rodas M., Valenzuela A. (2017). Docosahexaenoic Acid (DHA), a Fundamental Fatty Acid for the Brain: New Dietary Sources. Prostaglandins Leukot. Essent. Fat. Acids.

[52] Díaz M., Fabelo N., Ferrer I., Marín R. (2018). “Lipid raft aging” in the human frontal cortex during nonpathological aging: gender influences and potential implications in Alzheimer’s disease. Neurobiol. Aging.

[53] Cutuli D. (2017). Functional and Structural Benefits Induced by Omega-3 Polyunsaturated Fatty Acids During Aging. Curr. Neuropharmacol..

[54] Cabré R., Naudí A., Dominguez-Gonzalez M., Jové M., Ayala V., Mota-Martorell N., Pradas I., Nogueras L., Rué M., Portero-Otín M. (2018). Lipid Profile in Human Frontal Cortex Is Sustained Throughout Healthy Adult Life Span to Decay at Advanced Ages. J. Gerontol.—Ser. A Biol. Sci. Med. Sci..

[55] Castellani R.J., Rolston R.K., Smith M.A. (2010). Alzheimer disease. Disease-a-month DM.

[56] Rauk A. (2009). The Chemistry of Alzheimer’s Disease. Chem. Soc. Rev..

[57] John H., Gerald H. (1992). Alzheimer’ s Disease: The Amyloid Cascade Hypothesis. Science.

[58] Haass C., Selkoe D.J. (2007). Soluble Protein Oligomers in Neurodegeneration: Lessons from the Alzheimer’s Amyloid β-Peptide. Nat. Rev. Mol. Cell Biol..

[59] Walsh D.M., Selkoe D.J. (2007). Aβ Oligomers—A Decade of Discovery. J. Neurochem..

[60] Sakono M., Zako T. (2010). Amyloid Oligomers: Formation and Toxicity of Aβ Oligomers. FEBS J..

[61] Osborn L.M., Kamphuis W., Wadman W.J., Hol E.M. (2016). Progress in Neurobiology Astrogliosis: An Integral Player in the Pathogenesis of Alzheimer’s Disease. Prog. Neurobiol..

[62] Heneka M.T., Carson M.J., Khoury J.E., Landreth G.E., Brosseron F., Feinstein D.L., Jacobs A.H., Wyss-coray T., Vitorica J., Ransohoff R.M. (2015). Neuroinflammation in Alzheimer’s Disease. Lancet Neurol..

[63] Huang W., Zhang X., Chen W. (2016). Role of Oxidative Stress in Alzheimer’s Disease (Review). Biomed. Rep..

[64] Sisodia S.S., Gallagher M. (1998). A Role for the β-Amyloid Precursor Protein in Memory?. PNAS.

[65] Supnet C., Bezprozvanny I. (2010). The Dysregulation of Intracellular Calcium in Alzheimer Disease. Cell Calcium.

[66] Area-Gomez E., Schon E.A., Tagaya M., Simmen T. (2017). Alzheimer Disease. Organelle Contact Sites: From Molecular Mechanism to Disease.

[67] Ullah G., Demuro A., Parker I., Pearson J.E. (2015). Analyzing and Modeling the Kinetics of Amyloid Beta Pores Associated with Alzheimer’s Disease Pathology. PLoS ONE.

[68] Allinson T.M.J., Parkin E.T., Turner A.J., Hooper N.M. (2003). ADAMs Family Members as Amyloid Precursor Protein α-Secretases. 2003, 74, 342–352.

[69] Zhou Z.D., Chan C.H.S., Ma Q.H., Xu X.H., Xiao Z.C., Tan E.K. (2011). The Roles of Amyloid Precursor Protein (APP) in Neurogenesis, Implications to Pathogenesis and Therapy of Alzheimer Disease (AD). Cell Adhes. Migr..

[70] Parkin E.T., Watt N.T., Hussain I., Eckman E.A., Eckman C.B., Manson J.C., Baybutt H.N., Turner A.J., Hooper N.M. (2007). Cellular Prion Protein Regulates Beta-Secretase Cleavage of the Alzheimer’s Amyloid Precursor Protein. Proc. Natl. Acad. Sci. USA.

[71] Cheng H., Vetrivel K.S., Gong P., Meckler X., Parent A., Thinakaran G. (2007). Mechanisms of Disease: New Therapeutic Strategies for Alzheimer’s Disease—Targeting APP Processing in Lipid Rafts. Nat. Clin. Pract. Neurol..

[72] Lemkul J.A., Bevan D.R. (2011). Lipid Composition Influences the Release of Alzheimer’s Amyloid β-Peptide from Membranes. Protein Sci..

[73] Ikeda K., Yamaguchi T., Fukunaga S., Hoshino M., Matsuzaki K. (2011). Mechanism of Amyloid β-Protein Aggregation Mediated by GM1 Ganglioside Clusters. Biochemistry.

[74] Tong B.C.K., Wu A.J., Li M., Cheung K.H. (2018). Calcium Signaling in Alzheimer’s Disease & Therapies. Biochim. Biophys. Acta—Mol. Cell Res..

[75] Simons M., Keller P., De Strooper B., Beyreuther K., Dotti C.G., Simons K. (1998). Cholesterol Depletion Inhibits the Generation of Beta-Amyloid in Hippocampal Neurons. Proc. Natl. Acad. Sci. USA.

[76] Sparks D.L. (2007). Cholesterol Metabolism and Brain Amyloidosis: Evidence for a Role of Copper in the Clearance of Abeta through the Liver. Curr. Alzheimer Res..

[77] Fahrenholz F., Kojro E., Gimpl G., Lammich S., Ma W. (2001). Low Cholesterol Stimulates the Nonamyloidogenic Pathway by Its Effect on the α-Secretase ADAM 10. Proc. Natl. Acad. Sci. USA.

[78] Wahrle S., Das P., Nyborg A.C., McLendon C., Shoji M., Kawarabayashi T., Younkin L.H., Younkin S.G., Golde T.E. (2002). Cholesterol-Dependent γ-Secretase Activity in Buoyant Cholesterol-Rich Membrane Microdomains. Neurobiol. Dis..

[79] Marquer C., Devauges V., Cossec J., Duyckaerts C., Le S. (2011). Local Cholesterol Increase Triggers Amyloid Precursor Protein-Bace1 Clustering in Lipid Rafts and Rapid Endocytosis. FASEB J..

[80] Beel A.J., Sakakura M., Barrett P.J., Sanders C.R. (2010). Direct Binding of Cholesterol to the Amyloid Precursor Protein: An Important Interaction in Lipid-Alzheimer’s Disease Relationships?. Biochim. Biophys. Acta—Mol. Cell Biol. Lipids.

[81] Mayeux R. (2003). Epidemiology of Neurodegeneration. Annu. Rev. Neurosci..

[82] Cutler R.G., Kelly J., Storie K., Pedersen W.A., Tammara A., Hatanpaa K., Troncoso J.C., Mattson M.P. (2004). Involvement of Oxidative Stress-Induced Abnormalities in Ceramide and Cholesterol Metabolism in Brain Aging and Alzheimer’s Disease. Proc. Natl. Acad. Sci. USA.

[83] Sparks D.L. (1997). Coronary Artery Disease, Hypertension, ApoE, and Cholesterol: A Link to Alzheimer’s Disease?. Ann. N. Y. Acad. Sci..

[84] Vance J.E. (2014). MAM (Mitochondria-Associated Membranes) in Mammalian Cells: Lipids and Beyond. Biochim. Biophys. Acta—Mol. Cell Biol. Lipids.

[85] Tambini M.D., Pera M., Kanter E., Yang H., Guardia-Laguarta C., Holtzman D., Sulzer D., Area-Gomez E., Schon E.A. (2016). ApoE4 Upregulates the Activity of Mitochondria-Associated ER Membranes. EMBO Rep..

[86] Vaya J., Schipper H.M. (2007). Oxysterols, Cholesterol Homeostasis, and Alzheimer Disease. J. Neurochem..

[87] Marwarha G., Raza S., Prasanthi J.R.P., Ghribi O. (2013). Gadd153 and NF-ΚB Crosstalk Regulates 27-Hydroxycholesterol-Induced Increase in BACE1 and β-Amyloid Production in Human Neuroblastoma SH-SY5Y Cells. PLoS ONE.

[88] Brown J., Theisler C., Silberman S., Magnuson D., Gottardi-Littell N., Lee J.M., Yager D., Crowley J., Sambamurti K., Rahman M.M. (2004). Differential Expression of Cholesterol Hydroxylases in Alzheimer’s Disease. J. Biol. Chem..

[89] Bieschke J.A.N., Zhang Q., Bosco D.A., Lerner R.A., Powers E.T., Wentworth P., Kelly J.W. (2006). Small Molecule Oxidation Products Trigger Disease-Associated Protein Misfolding. Acc. Chem. Res..

[90] Famer D., Meaney S., Mousavi M., Nordberg A., Björkhem I., Crisby M. (2007). Regulation of α- and β-Secretase Activity by Oxysterols: Cerebrosterol Stimulates Processing of APP via the α-Secretase Pathway. Biochem. Biophys. Res. Commun..

[91] Hao M., Mukherjee S., Maxfield F.R. (2001). Cholesterol Depletion Induces Large Scale Domain Segregation in Living Cell Membranes. Proc. Natl. Acad. Sci. USA.

[92] Mondal M., Mesmin B., Mukherjee S., Maxfield F.R. (2009). Sterols Are Mainly in the Cytoplasmic Leaflet of the Plasma Membrane and the Endocytic Recycling Compartment in CHO Cells. Mol. Biol. Cell.

[93] Cecchi C., Nichino D., Zampagni M., Bernacchioni C., Evangelisti E., Pensal A., Liguri G., Gliozzi A., Stefani M., Relini A. (2009). A Protective Role for Lipid Raft Cholesterol against Amyloid-Induced Membrane Damage in Human Neuroblastoma Cells. Biochim. Biophys. Acta—Mol. Cell Biol. Lipids.

[94] Han X., Rozen S., Boyle S.H., Hellegers C., Cheng H., Burke J.R., Welsh-Bohmer K.A., Doraiswamy P.M., Kaddurah-Daouk R. (2011). Metabolomics in Early Alzheimer’s Disease: Identification of Altered Plasma Sphingolipidome Using Shotgun Lipidomics. PLoS ONE.

[95] Soreghan B., Thomas S.N., Yang A.J. (2003). Aberrant Sphingomyelin/Ceramide Metabolic-Induced Neuronal Endosomal/Lysosomal Dysfunction: Potential Pathological Consequences in Age-Related Neurodegeneration. Adv. Drug Deliv. Rev..

[96] Farooqui A.A. (2012). Lipid Mediators and Their Metabolism in the Nucleus: Implications for Alzheimer’s Disease. J. Alzheimer’s Dis..

[97] Ditaranto-Desimone K., Saito M., Tekirian T.L., Saito M., Berg M., Dubowchik G., Soreghan B., Thomas S., Marks N., Yang A.J. (2003). Neuronal Endosomal/Lysosomal Membrane Destabilization Activates Caspases and Induces Abnormal Accumulation of the Lipid Secondary Messenger Ceramide. Brain Res. Bull..

[98] Frisardi V., Panza F., Seripa D., Farooqui T., Farooqui A.A. (2011). Glycerophospholipids and Glycerophospholipid-Derived Lipid Mediators: A Complex Meshwork in Alzheimer’s Disease Pathology. Prog. Lipid Res..

[99] Haughey N.J., Bandaru V.V.R., Bae M., Mattson M.P. (2010). Roles for Dysfunctional Sphingolipid Metabolism in Alzheimer’s Disease Neuropathogenesis. Biochim. Biophys. Acta—Mol. Cell Biol. Lipids.

[100] Mielke M., Haughey N. (2013). Could Plasma Sphingolipids Be Diagnostic or Prognostic Biomarkers for Alzheimer’s Disease?. Clin. Lipidol..

[101] Furukawa K., Ohmi Y., Noriyo T., Yuji K., Orie T., Keiko F. (2011). Regulatory Mechanisms of Nervous Systems with Glycosphingolipids. Neurochem. Res..

[102] Wu G., Neil Z.L., Amin R., Ledeen R.W. (2011). Mice Lacking Major Brain Gangliosides Develop Parkinsonism. Neurochem. Res..

[103] Zha Q., Ruan Y., Hartmann T., Beyreuther K., Zhang D. (2004). GM1 Ganglioside Regulates the Proteolysis of Amyloid Precursor Protein. Mol. Psychiatry.

[104] Ariga T., Mcdonald M.P., Yu R.K. (2008). Role of Ganglioside Metabolism in the Pathogenesis of Alzheimer’s Disease—A Review. J. Lipid Res..

[105] Parton R.G. (1994). Ultrastructural Localization of Gangliosides; GM1 Is Concentrated in Caveolae. J. Histochem. Cytochem..

[106] Okada T., Ikeda K., Wakabayashi M., Ogawa M., Matsuzaki K. (2008). Formation of Toxic Aβ (1–40) Fibrils on GM1 Ganglioside-Containing Membranes Mimicking Lipid Rafts: Polymorphisms in Aβ (1–40) Fibrils. J. Mol. Biol..

[107] Zhang Q., Ohmi Y., Yamaguchi T., Furukawa K., Furukawa K., Yamauchi Y., Okajima T., Ohkawa Y. (2016). Expression of B4GALNT1, an Essential Glycosyltransferase for the Synthesis of Complex Gangliosides, Suppresses BACE1 Degradation and Modulates APP Processing. Sci. Rep..

[108] Posse de Chaves E., Sipione S. (2010). Sphingolipids and Gangliosides of the Nervous System in Membrane Function and Dysfunction. FEBS Lett..

[109] Ledeen R.W., Wu G. (2008). Nuclear Sphingolipids: Metabolism and Signaling. J. Lipid Res..

[110] Mocchetti I. (2005). Exogenous Gangliosides, Neuronal Plasticity and Repair, and the Neurotrophins. Cell. Mol. Life Sci..

[111] Saavedra L., Mohamed A., Kar S., De Chaves E.P. (2007). Internalization of Beta-Amyloid Peptide by Primary Neurons in the Absence of Apolipoprotein E. J. Biol. Chem..

[112] Fabelo N., Martín V., Marín R., Moreno D., Ferrer I., Díaz M. (2014). Altered Lipid Composition in Cortical Lipid Rafts Occurs at Early Stages of Sporadic Alzheimer’s Disease and Facilitates APP/BACE1 Interactions. Neurobiol. Aging.

[113] Navarro-Pardo E., Holland C.A., Cano A. (2018). Sex Hormones and Healthy Psychological Aging in Women. Front. Aging Neurosci..

[114] Mario D. (2016). Hippocampal Lipid Homeostasis in APP/PS1 Mice Is Modulated by a Complex Interplay Between Dietary DHA and Estrogens: Relevance for Alzheimer’s Disease. J. Alzheimer’s Dis..

[115] Canerina-Amaro A., Hernandez-Abad L.G., Ferrer I., Quinto-Alemany D., Mesa-Herrera F., Ferri C., Puertas-Avendano R.A., Diaz M., Marin R. (2017). Lipid Raft ER Signalosome Malfunctions in Menopause and Alzheimer’s Disease. Front. Biosci. (Schol. Ed.).

[116] Lees A.J., Hardy J., Revesz T. (2009). Parkinson’s Disease. Lancet.

[117] Broen M.P.G., Narayen N.E., Kuijf M.L., Dissanayaka N.N.W., Leentjens A.F.G. (2016). Prevalence of Anxiety in Parkinson’s Disease: A Systematic Review and Meta-Analysis. Mov. Disord..

[118] Ascherio A., Schwarzschild M.A. (2016). The Epidemiology of Parkinson’s Disease: Risk Factors and Prevention. Lancet Neurol..

[119] Reich S.G., Savitt J.M. (2019). Parkinson’s Disease. Med. Clin. N. Am..

[120] Hely M.A., Reid W.G.J., Adena M.A., Halliday G.M., Morris J.G.L. (2008). The Sydney Multicenter Study of Parkinson’s Disease: The Inevitability of Dementia at 20 Years. Mov. Disord..

[121] Pacheco C., Aguayo L.G., Opazo C. (2012). An Extracellular Mechanism That Can Explain the Neur Effects of α-Synuclein Aggregates in the Brain. Front. Physiol..

[122] Burré J. (2015). The Synaptic Function of α-Synuclein. J. Parkinson’s. Dis..

[123] Sharon R., Goldberg M.S., Bar-josef I., Betensky R.A., Shen J., Selkoe D.J. (2001). α-Synuclein Occurs in Lipid-Rich High Molecular Weight Complexes, Binds Fatty Acids, and Shows Homology to the Fatty Acid-Binding Proteins. Proc. Natl. Acad. Sci. USA.

[124] Colebc N.B., Murphy D.D., Grider T., Rueter S., Brasaemle D., Nussbaum R.L. (2002). Lipid Droplet Binding and Oligomerization Properties of the Parkinson’s Disease Protein α-Synuclein. J. Biol. Chem..

[125] Schoonenboom N.S.M., Mulder C., Vanderstichele H., Van Elk E.J., Kok A., Van Kamp G.J., Scheltens P., Blankenstein M.A. (2005). Effects of Processing and Storage Conditions on Amyloid β (1-42) and Tau Concentrations in Cerebrospinal Fluid: Implications for Use in Clinical Practice. Clin. Chem..

[126] Varkey J., Isas J.M., Mizuno N., Jensen M.B., Bhatia V.K., Jao C.C., Petrlova J., Voss J.C., Stamou D.G., Steven A.C. (2010). Membrane Curvature Induction and Tubulation Are Common Features of Synucleins and Apolipoproteins. J. Biol. Chem..

[127] Mizuno N., Varkey J., Kegulian N.C., Hegde B.G., Cheng N., Langen R., Steven A.C. (2012). Remodeling of Lipid Vesicles into Cylindrical Micelles by α-Synuclein in an Extended α-Helical Conformation. J. Biol. Chem..

[128] Westphal C.H., Chandra S.S. (2013). Monomeric Synucleins Generate Membrane Curvature. J. Biol. Chem..

[129] Shi Z., Sachs J., Rhoades E., Baumgart T., Haven N. (2015). Biophysics of α-Synuclein Induced Membrane Remodelling. Phys. Chem. Chem. Phys..

[130] Braun A.R., Lacy M.M., Ducas V.C., Rhoades E., Sachs J.N. (2014). A-Synuclein-Induced Membrane Remodeling Is Driven by Binding. J. Am. Chem. Soc..

[131] Madine J., Doig A.J., Middleton D.A. (2006). A Study of the Regional Effects of α-Synuclein on the Organization and Stability of Phospholipid Bilayers. Biochemistry.

[132] Payton J.E., Perrin R.J., Woods W.S., George J.M. (2004). Structural Determinants of PLD2 Inhibition by α-Synuclein. J. Mol. Biol..

[133] Gorbatyuk O.S., Li S., Nguyen F.N., Manfredsson F.P., Kondrikova G., Sullivan L.F., Meyers C., Chen W., Mandel R.J., Muzyczka N. (2009). α -Synuclein Expression in Rat Substantia Nigra Suppresses Phospholipase D2 Toxicity and Nigral Neurodegeneration. Mol. Ther..

[134] Vendruscolo M., Dobson C.M., Galvagnion C., Meisl G., Michaels T.C.T., Buell A.K., Knowles T.P.J. (2015). Lipid Vesicles Trigger α-Synuclein Aggregation by Stimulating Primary Nucleation. Nat. Chem. Biol..

[135] Nath S., Giraldo A.M.V., Eriksson I., Öllinger K., Bornefall P. (2017). Impact of High Cholesterol in a Parkinson’s Disease Model: Prevention of Lysosomal Leakage versus Stimulation of α-Synuclein Aggregation. Eur. J. Cell Biol..

[136] Cheng D., Kim W.S., Garner B. (2008). Regulation of α-Synuclein Expression by Liver X Receptor Ligands in Vitro. Neuroreport.

[137] Ghribi O., Schommer E., Feist G., Thomasson S., Thompson A., Rantham Prabhakara J.P. (2008). Differential Effects of 24-Hydroxycholesterol and 27-Hydroxycholesterol on Tyrosine Hydroxylase and α-Synuclein in Human Neuroblastoma SH-SY5Y Cells. J. Neurochem..

[138] Fan X., Kim H.-J., Parini P., Gabbi C., Warner M., Yakimchuk K., Gustafsson J.-A. (2008). Liver X Receptor (LXR): A Link between -Sitosterol and Amyotrophic Lateral Sclerosis-Parkinson’s Dementia. Proc. Natl. Acad. Sci. USA.

[139] Tansey M.G., Frank-Cannon T.C., Li H., Valasek M.A., Dietschy J.M., Turley S.D., Repa J.J. (2007). Liver X Receptor Activation Enhances Cholesterol Loss from the Brain, Decreases Neuroinflammation, and Increases Survival of the NPC1 Mouse. J. Neurosci..

[140] Hultenby K., Gustafsson J.-A., Wang L., Andersson S., Schuster G.U., Zhang Q. (2002). Liver X Receptors in the Central Nervous System: From Lipid Homeostasis to Neuronal Degeneration. Proc. Natl. Acad. Sci. USA.

[141] Barceló-Coblijn G., Golovko M.Y., Weinhofer I., Berger J., Murphy E.J. (2007). Brain Neutral Lipids Mass Is Increased in α-Synuclein Gene-Ablated Mice. J. Neurochem..

[142] Perrin R.J., Woods W.S., Clayton D.F., George J.M. (2002). Interaction of Human α-Synuclein and Parkinson’s Disease Variants with Phospholipids. J. Biol. Chem..

[143] Broersen K., Brink D.V.D., Fraser G., Goedert M., Davletov B. (2006). Alpha-Synuclein Adopts an Alpha-Helical Conformation in the Presence of Polyunsaturated Fatty Acids to Hinder Micelle Formation. Biochemistry.

[144] Sharon R., Bar-joseph I., Frosch M.P., Walsh D.M., Hamilton J.A., Selkoe D.J. (2003). The Formation of Highly Soluble Oligomers of Alpha-Synuclein Is Regulated by Fatty Acids and Enhanced in Parkinson’s Disease. Neuron.

[145] Lin P.Y., Huang S.Y., Su K.P. (2010). A Meta-Analytic Review of Polyunsaturated Fatty Acid Compositions in Patients with Depression. Biol. Psychiatry.

[146] El-Agnaf O.M.A., Jakes R., Curran M.D., Wallace A. (1998). Effects of the Mutations Ala30 to Pro and Ala53 to Thr on the Physical and Morphological Properties of α-Synuclein Protein Implicated in Parkinson’s Disease. FEBS Lett..

[147] Giasson B.I., Uryu K., Trojanowski J.Q., Lee V.M.Y. (1999). Mutant and Wild Type Human α-Synucleins Assemble into Elongated Filaments with Distinct Morphologies in Vitro. J. Biol. Chem..

[148] Pyszko J.A., Strosznajder J.B. (2014). Original Article the Key Role of Sphingosine Kinases in the Molecular Mechanism of Neuronal Cell Survival and Death in an Experimental Model of Parkinson’s Disease. Folia Neuropathol..

[149] Canerina-amaro A., Pereda D., Diaz M., Rodriguez-barreto D., Casañas-sánchez V., Heffer M., Garcia-esparcia P., Ferrer I., George S. (2019). Differential Aggregation and Phosphorylation of Alpha Synuclein in Membrane Compartments Associated with Parkinson Disease. Front. Neurosci..

[150] Martinez Z., Zhu M., Han S., Fink A.L. (2007). GM1 Specifically Interacts with Alpha-Synuclein and Inhibits Fibrillation. Biochem. Pharmacol..

[151] Ariga T. (2014). Pathogenic Role of Ganglioside Metabolism in Neurodegenerative Diseases. J. Neurosci. Res..

[152] Badawy M.M.S., Okada T., Kajimoto T., Hirase M., Matovelo S., Nakamura S., Yoshida D., Ijuin T., Nakamura S. (2018). Extracellular α-Synuclein Drives Sphingosine 1-Phosphate Receptor Subtype 1 out of Lipid Rafts, Leading to Impaired Inhibitory G-Protein Signaling. J. Biol. Chem..

[153] Fabelo N., Martin V., Santpere G., Marín R., Torrent L., Ferrer I., Díaz M. (2011). Severe Alterations in Lipid Composition of Frontal Cortex Lipid Rafts from Parkinson’s Disease and Incidental Parkinson’s Disease. Mol. Med..

[154] Davidson W.S., Jonas A., Clayton D.F., George J.M. (1998). Stabilization of Alpha-Synuclein Secondary Structure upon Binding to Synthetic Membranes. J. Biol. Chem..

[155] Kubo S., Nemani V.M., Chalkley R.J., Anthony M.D., Hattori N., Mizuno Y., Edwards R.H., Fortin D.L. (2005). A Combinatorial Code for the Interaction of Alpha-Synuclein with Membranes. J. Biol. Chem..

[156] Gedalya T.B., Loeb V., Israeli E., Altschuler Y., Selkoe D.J., Sharon R. (2009). α-Synuclein and Polyunsaturated Fatty Acids Promote Clathrin-Mediated Endocytosis and Synaptic Vesicle Recycling. Traffic.

[157] Madeira A., Yang J., Zhang X., Vikeved E., Nilsson A., Andrén P.E., Svenningsson P. (2011). Caveolin-1 Interacts with Alpha-Synuclein and Mediates Toxic Actions of Cellular Alpha-Synuclein Overexpression. Neurochem. Int..

[158] Fortin D.L. (2004). Lipid Rafts Mediate the Synaptic Localization of α-Synuclein. J. Neurosci..

[159] Ferrucci L., Giallauria F., Guralnik J.M. (2008). Epidemiology of Aging. Radiol. Clin. N. Am..

[160] Garn H., Coronel C., Waser M., Caravias G., Ransmayr G. (2017). Differential Diagnosis between Patients with Probable Alzheimer’s Disease, Parkinson’s Disease Dementia, or Dementia with Lewy Bodies and Frontotemporal Dementia, Behavioral Variant, Using Quantitative Electroencephalographic Features. J. Neural Transm..

[161] Jack C.R., Bennett D.A., Blennow K., Carrillo M.C., Dunn B., Haeberlein S.B., Holtzman D.M., Jagust W., Jessen F., Karlawish J. (2018). NIA-AA Research Framework: Toward a Biological Definition of Alzheimer’s Disease. Alzheimer’s Dement..

[162] Gibb W., Lees A.J. (1988). The Relevance of the Lewy Body to the Pathogenesis of Idiopathic Parkinson’s Disease. J. Neurol. Neurosurg. Psychiatry.

[163] Rao G., Eaton C. (2015). Does This Patient Have Parkinson Disease?. Mov. Disord..

[164] Tolosa E., Wenning G.W.P.W. (2006). The Diagnosis of Parkinson’s Disease. Lancet Neurol..

[165] DeMaagd G. (2016). Parkinson’s Disease and Its Management. Munic. Solid Waste Manag. Dev. Ctries..

[166] Han X., Fagan A.M., Cheng H., Morris J.C., Xiong C., Holtzman D.M. (2003). Cerebrospinal Fluid Sulfatide Is Decreased in Subjects with Incipient Dementia. Ann. Neurol..

[167] Mulder C., Wahlund L.O., Teerlink T., Blomberg M., Veerhuis R., Van Kamp G.J., Scheltens P., Scheffer P.G. (2003). Decreased Lysophosphatidylcholine/Phosphatidylcholine Ratio in Cerebrospinal Fluid in Alzheimer’s Disease. J. Neural Transm..

[168] Walter A., Korth U., Hilgert M., Hartmann J., Weichel O., Hilgert M., Fassbender K., Schmitt A., Klein J. (2004). Glycerophosphocholine Is Elevated in Cerebrospinal Fluid of Alzheimer Patients. Neurobiol. Aging.

[169] Mielke M.M., Haughey N.J., Bandaru V.V.R., Zetterberg H., Blennow K., Andreasson U., Johnson S.C., Gleason C.E., Blazel H.M., Puglielli L. (2014). Cerebrospinal Fluid Sphingolipids, β-Amyloid, and Tau in Adults at Risk for Alzheimer’s Disease. Neurobiol. Aging.

[170] Testa G., Staurenghi E., Zerbinati C., Gargiulo S., Iuliano L., Giaccone G., Fantò F., Poli G., Leonarduzzi G., Gamba P. (2016). Changes in Brain Oxysterols at Different Stages of Alzheimer’s Disease: Their Involvement in Neuroinflammation. Redox Biol..

[171] Heverin M., Bogdanovic N., Lütjohann D., Bayer T., Pikuleva I., Bretillon L., Diczfalusy U., Winblad B., Björkhem I. (2004). Changes in the Levels of Cerebral and Extracerebral Sterols in the Brain of Patients with Alzheimer’s Disease. J. Lipid Res..

[172] Catalá A., Díaz M. (2017). Impact of Lipid Peroxidation on the Physiology and Pathophysiology of Cell Membranes.

[173] Shinto L., Quinn J., Montine T., Dodge H.H., Woodward W., Baldauf-Wagner S., Waichunas D., Bumgarner L., Bourdette D., Silbert L. (2014). A Randomized Placebo-Controlled Pilot Trial of Omega-3 Fatty Acids and Alpha Lipoic Acid in Alzheimer’s Disease. J. Alzheimer’s Dis..

[174] Yuan L., Liu J., Ma W., Dong L., Wang W., Che R., Xiao R. (2016). Dietary Pattern and Antioxidants in Plasma and Erythrocyte in Patients with Mild Cognitive Impairment from China. Nutrition.

[175] Monacelli F., Borghi R., Cammarata S., Nencioni A., Piccini A., Tabaton M., Odetti P. (2015). Amnestic Mild Cognitive Impairment and Conversion to Alzheimer’s Disease: Insulin Resistance and Glycoxidation as Early Biomarker Clusters. J. Alzheimer’s Dis..

[176] Scheff S.W., Ansari M.A., Mufson E.J. (2016). Oxidative Stress and Hippocampal Synaptic Protein Levels in Elderly Cognitively Intact Individuals with Alzheimer’s Disease Pathology. Neurobiol. Aging.

[177] Rosén C., Mattsson N., Johansson P.M., Andreasson U., Wallin A., Hansson O., Johansson J.O., Lamont J., Svensson J., Blennow K. (2011). Discriminatory Analysis of Biochip-Derived Protein Patterns in CSF and Plasma in Neurodegenerative Diseases. Front. Aging Neurosci..

[178] Sepe F.N., Chiasserini D., Parnetti L. (2018). Role of FABP3 as Biomarker in Alzheimer’s Disease and Synucleinopathies. Future Neurol..

[179] Chiasserini D., Parnetti L., Andreasson U., Zetterberg H., Giannandrea D., Calabresi P., Blennow K. (2010). CSF Levels of Heart Fatty Acid Binding Protein Are Altered during Early Phases of Alzheimer’s Disease. J. Alzheimer’s Dis..

[180] Harari O., Chuchanga C., Pickering E.H., Bertelsen S., Fagan A.M., Holtzman D.M. (2015). Phosphorylated Tau-Aβ42 Ratio as a Continuous Trait for Biomarker Discovery for Early-Stage Alzheimer’s Disease in Multiplex Immunoassay Panels of Cerebrospinal Fluid. Biol. Psychiatry.

[181] Chaudhuri K.R., Martinez-Martin P. (2008). Quantitation of Non-Motor Symptoms in Parkinson’s Disease. Eur. J. Neurol..

[182] Braak H., Del Tredici K. (2008). Reply to “Controversies over the Staging of α-Synuclein Pathology in Parkinson’s Disease”. Acta Neuropathol..

[183] Mollenhauer B., Zhang J. (2012). Biochemical Premotor Biomarkers for Parkinson’s Disease. Mov. Disord..

[184] Mollenhauer B., Locascio J.J., Schulz-Schaeffer W., Sixel-Döring F., Trenkwalder C., Schlossmacher M.G. (2011). α-Synuclein and Tau Concentrations in Cerebrospinal Fluid of Patients Presenting with Parkinsonism: A Cohort Study. Lancet Neurol..

[185] Woulfe J.M., Duke R., Middeldorp J.M., Stevens S., Vervoort M., Hashimoto M., Masliah E., Chan P., Monte D.A.D., Langston J.W. (2002). Absence of Elevated Anti-Alpha-Synuclein and Anti-EBV Latent Membrane Protein Antibodies in PD. Neurology.

[186] Öhrfelt A., Grognet P., Andreasen N., Wallin A., Vanmechelen E., Blennow K., Zetterberg H. (2009). Cerebrospinal Fluid α-Synuclein in Neurodegenerative Disorders-A Marker of Synapse Loss?. Neurosci. Lett..

[187] Tateno F., Sakakibara R., Kawai T. (2012). Alpha-Synuclein in the Cerebrospinal Fluid Differentiates Synucleinopathies (Parkinson Disease, Dementia with Lewy Bodies, Multiple System Atrophy) from Alzheimer Disease. Alzheimer Dis. Assoc. Disord..

[188] Park M.J., Cheon S.M., Bae H.R., Kim S.H., Kim J.W. (2011). Elevated Levels of α-Synuclein Oligomer in the Cerebrospinal Fluid of Drug-Naïve Patients with Parkinson’s Disease. J. Clin. Neurol..

[189] Parnetti L., Chiasserini D., Bellomo G., Giannandrea D., de Carlo C., Qureshi M.M., Ardah M.T., Varghese S., Bonanni L., Borroni B. (2011). Cerebrospinal Fluid Tau/α-Synuclein Ratio in Parkinson’s Disease and Degenerative Dementias. Mov. Disord..

[190] Wang Y., Shi M., Chung K.A., Zabetian C.P., Leverenz J.B., Berg D., Srulijes K., Trojanowski J.Q., Lee V.M.Y., Siderowf A.D. (2012). Phosphorylated α-Synuclein in Parkinson’s Disease. Sci. Transl. Med..

[191] Kang J.H., Irwin D.J., Chen-Plotkin A.S., Siderowf A., Caspell C., Coffey C.S., Waligórska T., Taylor P., Pan S., Frasier M. (2013). Association of Cerebrospinal Fluid β-Amyloid 1-42, t-Tau, p-Tau181, and α-Synuclein Levels with Clinical Features of Drug-Naive Patients with Early Parkinson Disease. JAMA Neurol..

[192] Wennström M., Surova Y., Hall S., Nilsson C., Minthon L., Boström F., Hansson O., Nielsen H.M. (2013). Low CSF Levels of Both α-Synuclein and the α-Synuclein Cleaving Enzyme Neurosin in Patients with Synucleinopathy. PLoS ONE.

[193] Parnetti L., Castrioto A., Chiasserini D., Persichetti E., Tambasco N., El-agnaf O., Calabresi P. (2013). Cerebrospinal Fluid Biomarkers in Parkinson Disease. Nat. Rev. Neurol..

[194] Parnetti L., Farotti L., Eusebi P., Chiasserini D., De Carlo C., Giannandrea D., Salvadori N., Lisetti V., Tambasco N., Rossi A. (2014). Differential Role of CSF Alpha-Synuclein Species, Tau, and Aβ42 in Parkinson’s Disease. Front. Aging Neurosci..

[195] Van Dijk K.D., Persichetti E., Chiasserini D., Eusebi P., Beccari T., Calabresi P., Berendse H.W., Parnetti L., van de Berg W.D.J. (2013). Changes in Endolysosomal Enzyme Activities in Cerebrospinal Fluid of Patients with Parkinson’s Disease. Mov. Disord..

[196] Aerts M.B., Esselink R.A.J., Abdo W.F., Bloem B.R., Verbeek M.M. (2012). CSF α-Synuclein Does Not Differentiate between Parkinsonian Disorders. Neurobiol. Aging.

[197] He R., Yan X., Guo J., Xu Q., Tang B., Sun Q. (2018). Recent Advances in Biomarkers for Parkinson’s Disease. Front. Aging Neurosci..

[198] Concannon R., Finn D.P., Dowd E. (2015). Cannabinoids in Parkinson’s Disease. Cannabinoids Neurol. Ment. Dis..

[199] Pisani A., Fezza F., Galati S., Battista N., Napolitano S., Finazzi-Agrò A., Bernardi G., Brusa L., Pierantozzi M., Stanzione P. (2005). High Endogenous Cannabinoid Levels in the Cerebrospinal Fluid of Untreated Parkinson’s Disease Patients. Ann. Neurol..

[200] Pisani V., Moschella V., Bari M., Fezza F., Galati S., Bernardi G., Stanzione P., Pisani A., Maccarrone M. (2010). Dynamic Changes of Anandamide in the Cerebrospinal Fluid of Parkinson’s Disease Patients. Mov. Disord..

[201] Emamzadeh F.N. (2017). Role of Apolipoproteins and α-Synuclein in Parkinson’s Disease. J. Mol. Neurosci..

[202] Elliott D.A., Weickert C.S., Garner B. (2010). Apolipoproteins in the Brain: Implications for Neurological and Psychiatric Disorders. Clin. Lipidol..

[203] Qiang J.K., Wong Y.C., Siderowf A., Hurtig H.I., Xie S.X., Lee V.M., Trojanowski J.Q., Yearout D., Leverenz J., Thomas J. (2013). Plasma Apolipoprotein A1 as a Biomarker for Parkinson’s Disease. Ann. Neurol..

[204] Swanson C.R., Berlyand Y., Xie S.X., Alcalay R.N., Chahine L.M., Chen-Plotkin A.S. (2015). Plasma Apolipoprotein A1 Associates with Age at Onset and Motor Severity in Early Parkinson’s Disease Patients. Mov. Disord..

[205] Chalimoniuk M., Snoek G.T., Adamczyk A., Małecki A., Strosznajder J.B. (2006). Phosphatidylinositol Transfer Protein Expression Altered by Aging and Parkinson Disease. Cell. Mol. Neurobiol..

[206] Dexter D.T., Carter C.J., Wells F.R., Javoy-Agid F., Agid Y., Lees A., Jenner P., Marsden C.D. (1989). Basal Lipid Peroxidation in Substantia Nigra Is Increased in Parkinson’s Disease. J. Neurochem..

[207] Yoritaka A., Uchida K., Stadtman E.R., Hattori N., Tanaka M., Mizuno Y. (1996). Immunohistochemical Detection of 4-Hydroxynonenal Protein Adducts in Parkinson Disease. Proc. Natl. Acad. Sci. USA.

[208] Gmitterova K., Heinemann U., Gawinecka J., Varges D., Ciesielczyk B., Valkovic P., Benetin J., Zerr I. (2009). 8-OHdG in Cerebrospinal Fluid as a Marker of Oxidative Stress in Various Neurodegenerative Diseases. Neurodegener. Dis..

[209] Isobe C., Abe T., Terayama Y. (2010). Levels of Reduced and Oxidized Coenzyme Q-10 and 8-Hydroxy-2′- Deoxyguanosine in the CSF of Patients with Alzheimer’s Disease Demonstrate That Mitochondrial Oxidative Damage and/or Oxidative DNA Damage Contributes to the Neurodegenerative Process. J. Neurol..

[210] Kikuchi A., Takeda A., Onodera H., Kimpara T., Hisanaga K., Sato N., Nunomura A., Castellani R.J., Perry G., Smith M.A. (2002). Systemic Increase of Oxidative Nucleic Acid Damage in Parkinson’s Disease and Multiple System Atrophy. Neurobiol. Dis..

[211] He X., Huang Y., Li B., Gong C.X., Schuchman E.H. (2010). Deregulation of Sphingolipid Metabolism in Alzheimer’s Disease. Neurobiol. Aging.

[212] González-Domínguez R., García-Barrera T., Gómez-Ariza J.L. (2014). Combination of Metabolomic and Phospholipid-Profiling Approaches for the Study of Alzheimer’s Disease. J. Proteom..

[213] Yanai H. (2017). Effects of N-6 and n-3 Polyunsaturated Fatty Acids on Colorectal Carcinogenesis. J. Clin. Med. Res..

[214] Thomas H., Pelleieux S., Vitale N., Oliver J. (2016). Arachidonic Acid in Alzheimer’s Disease. J. Neurol. Neuromed..

[215] Fraser T., Tayler H., Love S. (2010). Fatty Acid Composition of Frontal, Temporal and Parietal Neocortex in the Normal Human Brain and in Alzheimer’s Disease. Neurochem. Res..

[216] Patel D., Witt S.N. (2017). Ethanolamine and Phosphatidylethanolamine: Partners in Health and Disease. Oxid. Med. Cell. Longev..

[217] Farmer K., Smith C.A., Hayley S., Smith J. (2015). Major Alterations of Phosphatidylcholine and Lysophosphotidylcholine Lipids in the Substantia Nigra Using an Early Stage Model of Parkinson’s Disease. Int. J. Mol. Sci..

